# Clinical significance and mechanisms associated with segmental UPD

**DOI:** 10.1186/s13039-021-00555-0

**Published:** 2021-07-20

**Authors:** Peter R. Papenhausen, Carla A. Kelly, Samuel Harris, Samantha Caldwell, Stuart Schwartz, Andrea Penton

**Affiliations:** 1 Cytogenetics Department, Laboratory Corporation of America, Research Triangle Park, NC 27709 USA; 2 Morsani College of Medicine, University of South Florida, Tampa, FL 33612 USA

**Keywords:** Uniparental disomy, Segmental uniparental disomy, Mitotic correction, Chromosome microarray (CMA), Cytogenetics, Homologous recombination

## Abstract

Whole chromosome uniparental disomy (UPD) has been well documented with mechanisms largely understood. However, the etiology of segmental limited UPD (segUPD) is not as clear. In a 10-year period of confirming (> 300) cases of whole chromosome UPD, we identified 86 segmental cases in both prenatal and postnatal samples. Thirty-two of these cases showed mosaic segmental UPD at 11p due to somatic selection associated with Beckwith–Wiedemann syndrome. This study focuses on apparent mechanisms associated with the remaining cases, many of which appear to represent corrections of genomic imbalance such as deletions and derivative chromosomes. In some cases, segmental UPD was associated with the generation of additional genomic imbalance while in others it apparently resulted in restoration of euploidy. Multiple tests utilizing noninvasive prenatal testing (NIPT), chorionic villus sampling (CVS) and amniotic fluid samples from the same pregnancy revealed temporal evidence of correction and a “hotspot” at 1p. Although in many cases the genomic imbalance was dosage “repaired” in the analyzed tissue, clinical effects could be sustained due to early developmental effects of the original imbalance or due to its continued existence in other tissues. In addition, if correction did not occur in the gametes there would be recurrence risks for the offspring of those individuals. Familial microarray allele patterns are presented that differentiate lack of gamete correction from somatic derived gonadal mosaicism. These results suggest that the incidence of segUPD mediated correction is underestimated and may explain the etiology of some clinical phenotypes which are undetected by routine microarray analysis and many exome sequencing studies.

**Supplementary Information:**

The online version contains supplementary material available at 10.1186/s13039-021-00555-0.

## Background

Whole chromosome uniparental disomy (UPD), the inheritance of both homologues from one parent, has been confirmed as primarily originating from somatic corrections of meiotically-derived trisomy or monosomy [1]. Uniparental heterodisomy is the inheritance of different chromosome homologues from one parent, while uniparental isodisomy is the inheritance of two copies of a single chromosome homologue from one parent. Individuals with mixed UPD (mixUPD) have stretches of isodisomic segments and heterodisomic segments because of meiotic recombination. Isodisomic segments are visible on SNP microarrays as extended runs of homozygosity (ROH) and, in the absence of other chromosomes with extended ROH due to consanguinity, the isodisomic segments provide a distinct clue to the presence of UPD [2, 3].

Identification of UPD is clinically important because it can be accompanied by imprinting disorders, autosomal recessive disorders, or aberrant development due to the presence of transient aneusomy [4, 5]. The incidence of whole chromosome UPD has been estimated at 1 in 3500 newborns [6], but more recent molecular studies estimate the prevalence to be much greater at about 0.2–0.3% [7–9]. In contrast, reports of copy-neutral segmentally-restricted UPD are much rarer at about 0.03%, excluding Beckwith–Wiedemann syndrome (BWS) cases associated with imprinted paternal growth advantage at 11p15.5 [10, 11].

**Fig. 1:**
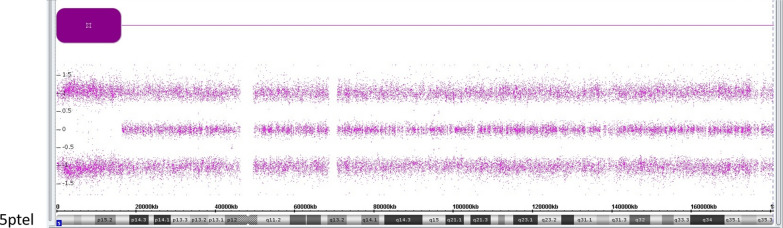
CMA analysis case example of possible segUPD. A > 16 Mb terminal ROH at 5p in case 7 depicted by the purple block with much shorter ROH elsewhere in the analysis inconsistent with a consanguinity correlation. Parental follow-up confirmed maternal segUPD

SegUPD is especially common in cancer and, in those studies, it is generally referred to as copy-neutral loss of heterozygosity (CN-LOH). The clonal evolution process resulting in CN-LOH is based on selective proliferative advantage, primarily driven by loss of tumor suppressor genes or acquisition of homozygosity for oncogenic mutations [2]. Constitutional segUPD is typically detected in single nucleotide polymorphism (SNP) chromosomal microarray analysis (CMA) as a long terminal ROH in a single chromosome (Fig. 1) that, in molecular follow-up, shows allelic exclusions from one parent in the ROH and biparental allele inheritance in the rest of the chromosome. Consanguinity can present a diagnostic problem for detection of segUPD, but is associated with ROH in multiple chromosomes that are usually not found exclusively in terminal regions [12]. The American College of Medical Genetics has proposed guidelines for diagnostic testing of suspected whole chromosome UPD and segUPD [13].

**Fig. 2:**
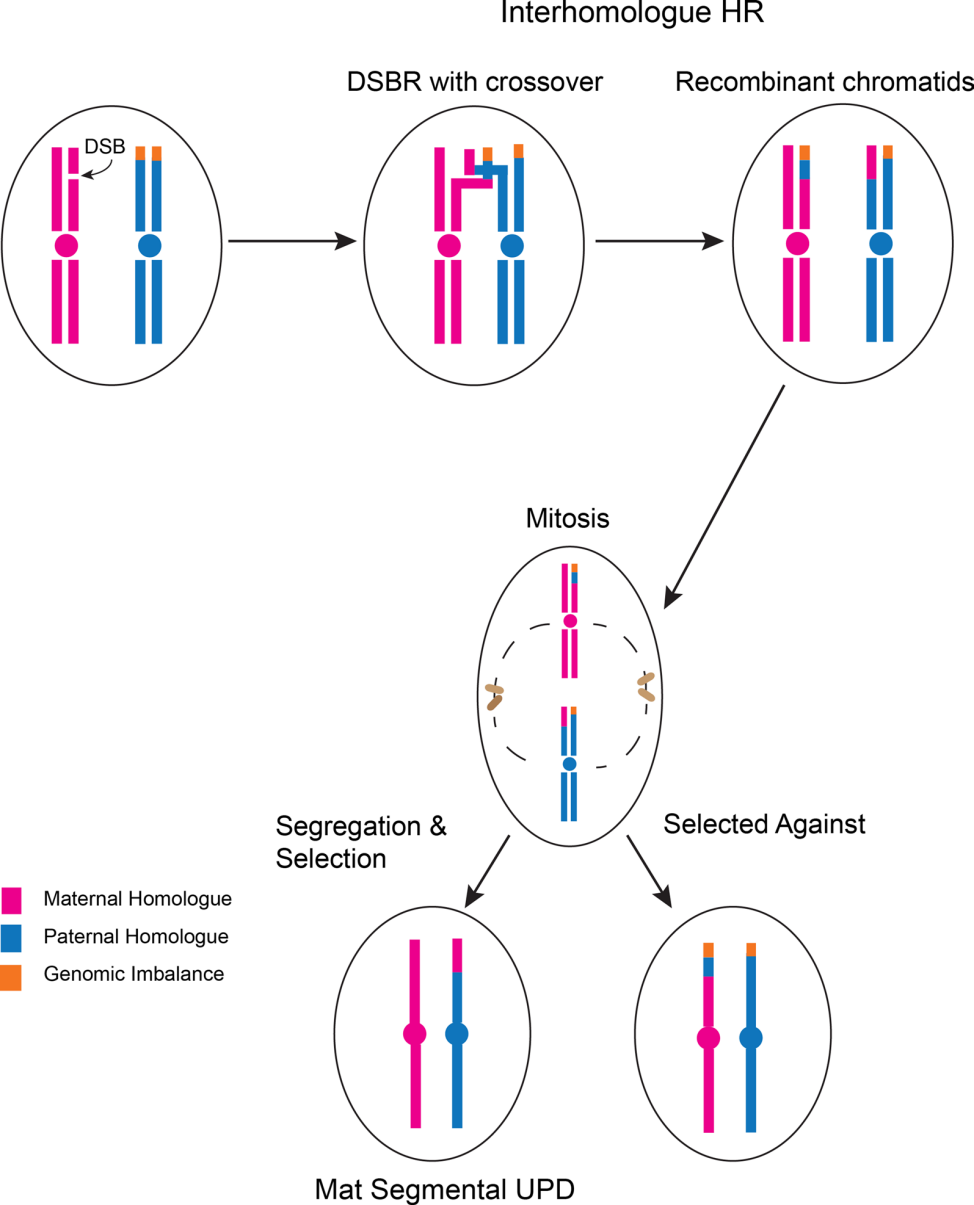
Graphic depiction of mitotic-mediated correction of meiotically derived imbalance. Double-strand break mediated repair results in inter-homologue recombination during S phase of a diploid chromatid (pink) with a chromatid (blue) containing a terminal rearrangement (orange). This results in two chromosome homologues, each containing one recombinant chromatid. Subsequent mitotic segregation can result in two outcomes: two daughter cells that, like the parent cell, are heterozygous for the original imbalance or in the outcome described in the figure. In this case one daughter cell is homozygous for the imbalance and the other is euploid but with uniparental inheritance in the recombinant region, segUPD, detected as a terminal ROH in microarray testing. Survival and expansion are more likely for the euploid daughter cell. However, variable selective pressure, both in cancer and some constitutional alterations, can result in clonal expansion of cells with the imbalance post mitotic recombination

The mechanism of origin of segUPD is different from that of whole chromosome UPD and thought to arise from homologous recombination due to interhomologue repair of double-stranded DNA breaks that occur somatically followed by subsequent clonal expansion of the recombinant cell [14] (Fig. 2). The homologue repair, thus, results in subpopulations with exclusive isoallelic segments that typically involve the terminal arm. DNA repair mechanisms that don’t generate crossovers can result in interstitial regions of segUPD. There is evidence for these instances of segUPD described in two studies involving chromosome 14 [15, 16] and in the UPD database [17]. Interstitial segUPD, however, has not been detected in the previously mentioned sequence analysis of trios or in the current study. This may be due to the fact that short interstitial regions of segUPD, expected with DNA repair involving no crossovers, are not detected by the present sequencing algorithms and CMA criteria. In addition, there may be lack of selective pressure for a second recombination event necessary for interstitial segUPD when a first recombination event has corrected an imbalance.

Using high resolution CMA in this study, we have identified results consistent with either segUPD or apparent mosaic segUPD over a 10-year period. Although all non-mosaic cases were molecularly confirmed, the presence of normal cells resulted in inconclusive results in mosaic cases. The etiology and associated clinical significance of segUPD is the focus of this report, along with the importance of appropriate genetic counseling.

## Materials and methods

DNA samples for microarray analysis were extracted either from placental cells, amniocytes, buccal cells or leukocytes using standard methods. DNA was digested with Nsp1, ligated to adaptors, and amplified using Titanium Taq with a GeneAmp PCR System 9700. PCR products were purified using AMPure beads and quantified using a NanoDrop 8000. Purified DNA was fragmented, biotin-labeled and hybridized to the Affymetrix 6.0^®^ or Cytoscan HD chip^®^ (Thermo Fisher Scientific). The two versions have ~ 1.8 million probes with ~ 50% SNP targeting and ~ 2.7 million probes with ~ 33% SNP targeting, respectively. The copy number state is indicated by either the Log2 signal intensity ratio tract (Log2(sample/reference signal)) or the Smooth signal tract, a Gaussian smoothed calibrated copy number estimate. SNP genotypes are determined by the allele difference tract. Each probe corresponds to a unique genomic position and is visualized as a point in the Log2 and allele difference tracts. Proprietary chromosome analysis suite software (CHAS) from Applied Biosytems was utilized for analysis of all microarray data in this study. The analysis was based on the GRCh37/hg19 assembly.

**Fig. 3:**
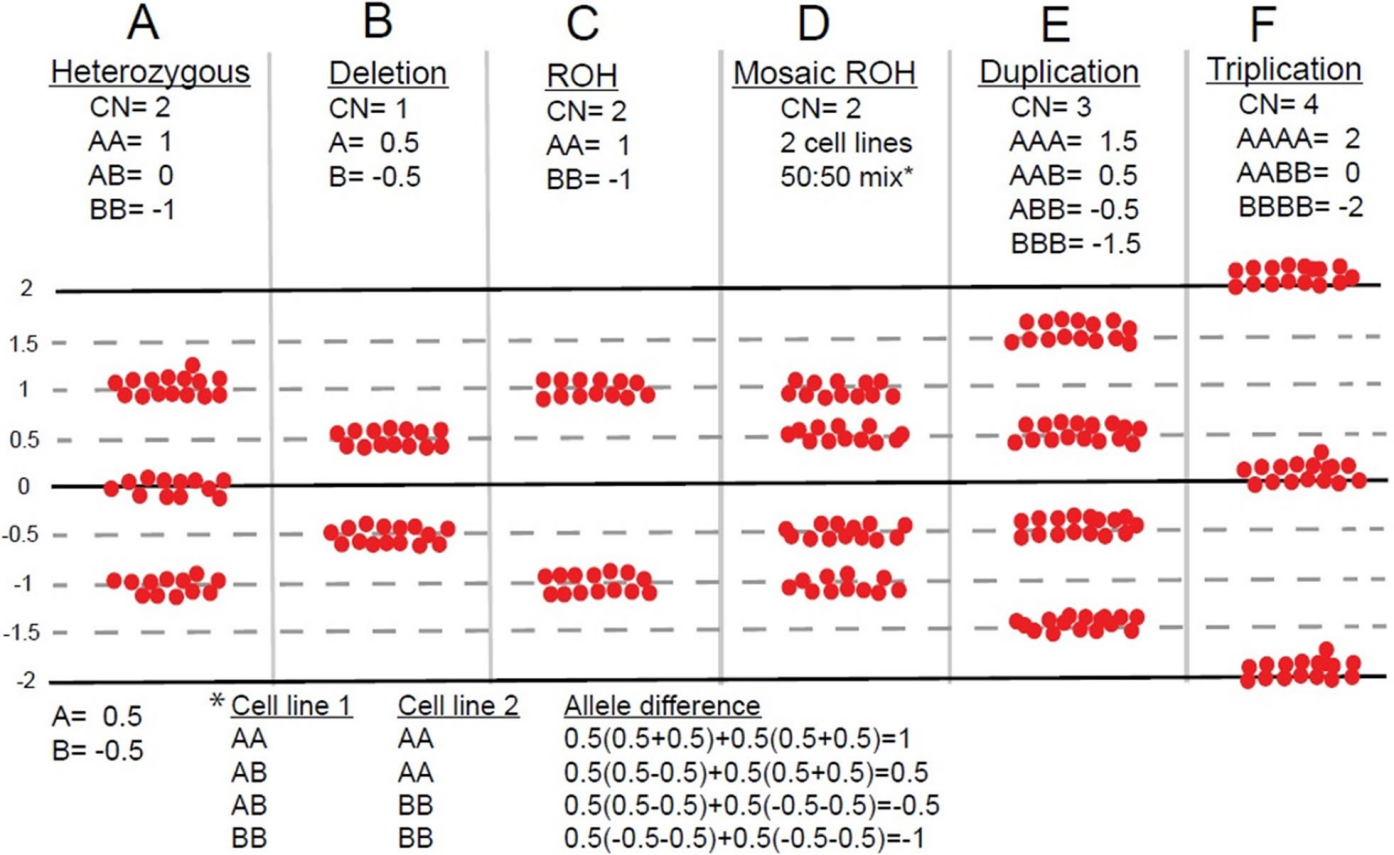
Graphic explanation of allele difference dosage plots in the Chromosome Analysis Suite (ChAS)^®^ SNP software. Each SNP at a given genomic position is assigned either an A or B designation (dependent on the polymorphic base pair at that location) with A = 0.5 and B = − 0.5 and the final value equal to the sum of alleles at a particular genomic position. **A** A heterozygous diploid allele mix consists of either two A alleles (AA) with a value of 1, one A allele and one B allele (AB) with a value of 0, or two B alleles (BB) with a value of -1 that result in the three tracts shown. **B** A deletion shows either an A or B allele and only two tracts at a value of 0.5 or − 0.5. **C** A run of copy neutral homozygosity consists of only the AA or BB allele pattern with a value of 1.0 or − 1.0. **D** A 50:50 mix of two cell lines in which cell line 1 is heterozygous at a certain position and cell line 2 is homozygous at that position. Note that the heterozygous alleles are shifted away from the midline due to the homozygous admixture. **E** A duplication shows an AAA, AAB, ABB or BBB (4 tract) pattern with an allele difference value of 1.5, 0.5, − 0.5 or − 1.5. **F** A triplication with 2 identical maternal and paternal copies results in an AAAA, AABB, and BBBB allele pattern with an allele difference value of 2.0, 0 or − 2.0 and three allele tracts. Typical triplications have four or five allele tracts

Based on laboratory empirical data and microarray validation studies, an extended (> 8 Mb) terminal ROH in a single chromosome is the established cutoff that warrants confirmation of potential whole chromosome UPD or segUPD. Parental follow-up with region specific microsatellites or by trio CMA allele comparisons differentiates between potential UPD or an isolated region of shared parental ancestry. Multiple (> 2) parental exclusions along the whole chromosome defines whole chromosome UPD, while exclusions restricted to the homozygous segment defines segUPD. Terminal regions with mosaic segUPD are distinctive because they show four tracts, with the distance of the two inner tracts from the central line defining the percentage of the homozygous copy-neutral cell line (Fig. 3). Mosaic cases are likely to represent segUPD, but due to the admixture of cells with biparental alleles and the non-quantitative nature of microsatellite UPD testing, confirmation testing was not attempted. Quantitative pyrosequencing may be used for cases in which the parent of origin allelic dosage ratios need to be confirmed, but was not readily available.

Non-invasive prenatal testing (NIPT), a prenatal screen that analyzes cell free DNA (cfDNA) fragments in maternal plasma derived from the placental trophoblast, was performed by genome-wide massive parallel sequencing (MPSS), as per Jensen, 2013 (Sequenom, a wholly owned subsidiary of Laboratory Corporation of America) [18]. The test is usually performed from 13 to 20 weeks gestation and, though often used as an aneuploidy screen, higher resolution was available, which allowed detection of deletions and duplications ≥ 7 Mb in the cases presented [19].

Fluorescence in situ hybridization (FISH), chromosome studies, and microsatellite analyses were performed using standard techniques. Appropriate informed consent was obtained from human subjects.

## Results

**Fig. 4:**
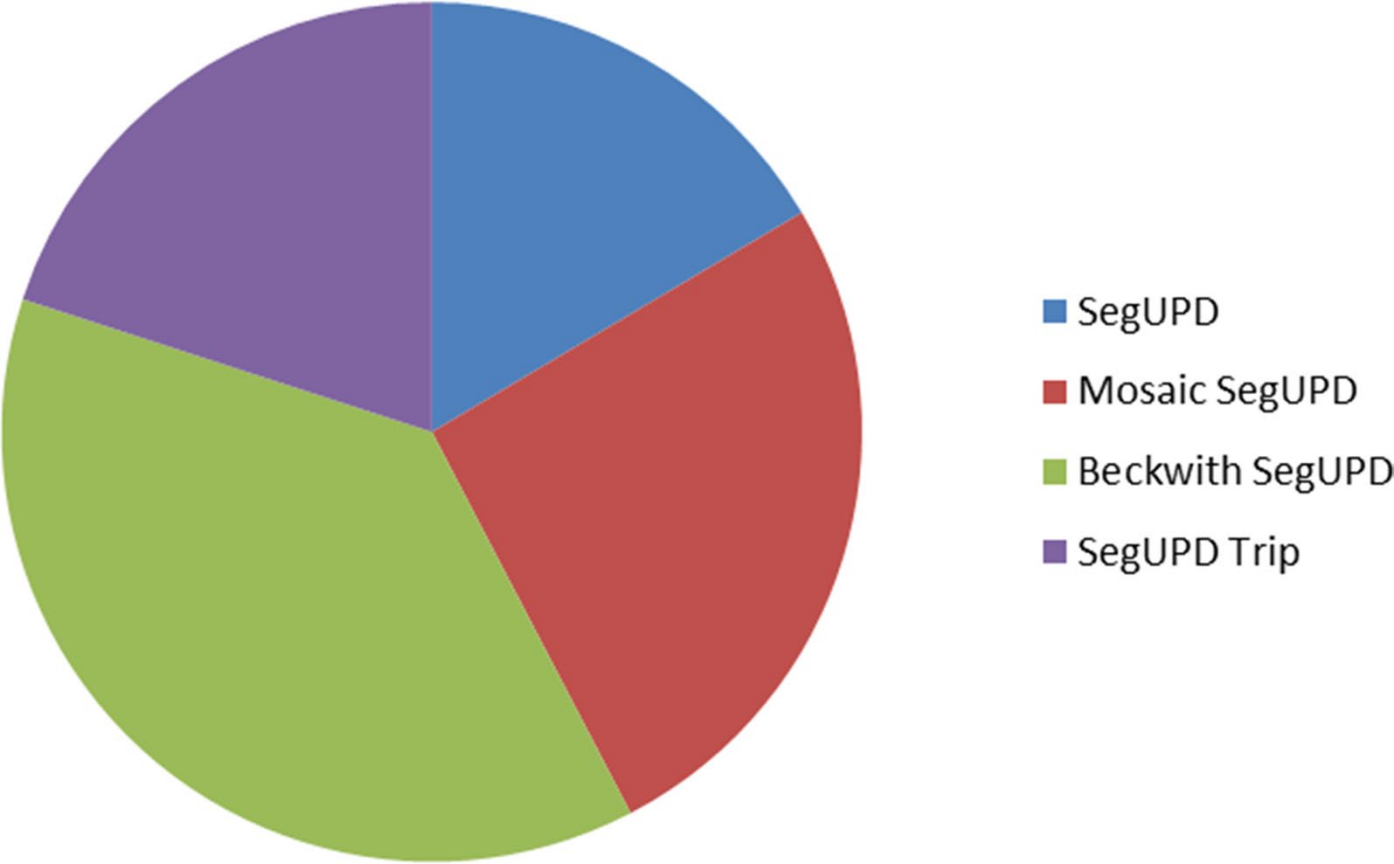
Relative incidence of segUPD subgroups seen during study timeframe

A cohort of 85 cases was compiled for this study. More than a third (32/85) of the cases of segUPD identified were associated with terminal mosaic ROH of various lengths and percentages on the p arm of chromosome 11 (Fig. 4), the phenotypes of which were consistent with BWS. The remaining 53 cases were grouped into three categories: those with non-mosaic terminal segUPD (14 cases—Table 1), those with mosaic terminal segUPD (22 cases—Table 2) and those with terminal segUPD contiguous with triplications (17 cases—Table 3).

**Table 1:** Segmental UPD

Case	UPD interval	TERMINAL ROH (Mb)	Origin	Age/source	Indication
1	1pter → p36.22	9.39	Mat	9.3 years	Multiple congenital anomalies: der(1)t(1;17) in amnio analysis
2	1pter → p36.22	9.33^a^	Mat	CVS and AF	NIPT: Terminal del(1)(p36), 60% deletion in CVS, copy neutral in AF, normal fetal ultrasound
3	1pter → p36.12	21.58	NA	AF	NIPT: Terminal del(1)(p36), terminal dup(1)(q42); cardiac defect, hypotonic, dysmorphic at birth
4	1pter → p36.13	16.32^b^	Mat	AF and POC	NIPT: Terminal del(1)(p36.23) and dup(1)(p36.23p36.22), normal pediatric follow-up
5	1pter → p36.13	17.28^c^	NA	Newborn and Placenta	NIPT: Terminal del(1)(p36.13) and mos dup(18)(q22q23), VSD, esophageal atresia
6	3pter → p22.1	43.13^d^	Mat	24 years blood and buccal	Developmental delay, hearing loss, obesity, mosaic unbalanced der(4)t(3;4)(p22;q35) detected in early childhood
7	5pter → p15.1	16.44	Mat	51 years	Cardiomyopathy, hypertension, mental retardation
8	7q33 → qter	26.21^e^	Mat	CVS and AF	Ventricular septal defect, small right ventricle
9	10q26.13 → qter	11.12	Mat	Newborn	Clinically normal; CVS with terminal G-band del(10)(q26)
10	11q13.1 → qter	70.88	Mat	12 years	Encephalopathy
11	14q24.3 → qter	32.21	Pat	AF	Scalp and leg edema, short femurs/thorax (UPD14pat related)
12	15q15.3 → qter	58.54	Pat	42 years	Recurrent pregnancy loss
13	Xq25 → qter	29.32	Mat	Newborn	Fragile X full mutation x2, no paternal repeat
14	Xq13.1 → qter	85.05	Pat	2 years	Congenital anomalies of face and neck

^a^Same ROH in both prenatal specimens; mosaic deletion in CVS, arr 1p36.33p36.22(849466–9292997)x1[0.3]

^b^Mosaic del/dup in post-delivery POC array analysis, arr 1p36.33p36.23(849466–8718884)x1[0.8], arr 1p36.23p36.22(8718885–12155862)x3[0.8]

^c^Non-mosaic deletion in POC, arr 1p36.33p36.13(849466–17275759)x1

^d^Mosaic gain in adult blood CMA (chromosome analysis confirmed), arr 3p26.3p22.1(2693–43129322)x3[0.05]

^e^Mosaic copy neutral ROH in CVS, arr 7q33q36.3(132913480–159119220)x2[0.5] hmz. Non-mosaic in AF

**Table 2:** Mosaic segmental UPD

Case	segUPD interval	TERMINAL ROH (Mb)	~ % Mosaic HMZ	Age/Source	Indication
15	1pter → p22.1	93.9	50	3 years	Developmental delay, seizures, hearing loss, decreased motor function
16	1pter → p36.32	2.8	34	1.9 mos	Seizures in infant, suspect epilepsy
17	1q12 → qter	99.4	50	1.5 years	Macrocephaly, delays in development and speech, short stature
18	1q42.13 → qter	20.2	15	CVS	AMA, family history of chromosome abnormality
19	3pter → p24.3	23.46	30	AF	AV canal defect, 2 vessel cord, co-twin with anencephaly, oligohdramnios
20	5pter → p13.2	36.85	50	CVS	Prenatal anxiety
21	9q13 → qter	74.2	40	4 years	Autism
22	9q13 → qter	74.2	20	POC	Loss at 22 weeks
23	11q13.4 → qter	62.34	25	AF	Clinodactyly, bright bowel
24	12q13.13 → qter	80.87	25 (blood); 15 (buccal); 0 (villi)	NB	Dysmorphic features, Mowat-Wilson syndrome (a pathogenic mutation in *ZEB2* was correlated with the phenotype)
25	12q13.11 → 12q13.13	84.36	70 85	1.5 years	Peg teeth, dry skin, developmental delay, speech and motor delay
	12q13.13 → qter				
26	13q12.3 → qter	85.59	20	5.3 years	Atrial septal defect
27	13q12.11 → qter	94.9	35	3.9 years	Essential (primary) hypertension, obesity, abnormal weight gain
28	14q12 → qter	74.68	25	8.9 years	Developmental delay
29	14q22.1 → qter	55.56	27	3.4 years	Autism, multiple congenital anomalies
30	15q13.3 → q15.2		15	12 years	Short stature, developmental delay; symptoms consistent with 15q25.2 microdeletion syndrome
	15q15.2 → q22.31	71.24	46		
	15q22.31 → qter		75^a^		
31	15q22.31 → qter	37.8	30	CVS	NT 3.9 mm
32	16q11.2 → qter	43.7	35	NB	Microcephaly, fetal growth restriction
33	18q11.1 → qter	59.5	25	POC	Positive MSS, increased risk of 4p deletion, features consistent with Wolf–Hirschhorn syndrome
34	19q13.2 → q13.2	2.75	25	35 years	Progressive progeria-like symptoms, short stature, psychoses
	19q13.2 → qter	17.36	37		
35	21q21.1 → qter	24.38	40^b^	29 years	Developmental disorder of scholastic skills
36	22q11.23 → qter	26.6	50	POC	Abnormal cfDNA screen with fetal demise

^a^Mosaic deletion, arr 15q25.1q25.3 (78989949–86981470)x1[0.25]

^b^Mosaic duplication, arr 12p13.33p11.22(173786–30511741)x3[0.6], and mosaic deletion, arr 21q22.2q22.3(41025556–48097372)x1[0.6]

**Table 3:** Segmental UPD contiguous with triplications

Case	UPD interval	Terminal ROH (Mb)	Triplication interval	Triplication size (Mb)	Age/source	Indication
37	1pter → p36.22	11.62	1p36.22 → p36.21	1.32	4.8 years	None given
38	1pter → p36.13	19.35	1p36.13 → p36.12	3.56	7.6 years	None given
39	1pter → p36.33	1.49	1p36.33 → p36.32	2.09	1.2 years	Developmental delay
40	1q43 → qter	10.07	1q42.3 → q43	4.23	NB	Multiple congenital anomalies
41	2pter → p24.2	17.48	2p24.2 → p23.3	8.24	CVS	Cystic hygroma
42	3pter → p25.1	13.65	3p25.1 → p24.1	14.7	4.9 years	Developmental delay
43	4pter → p15.2	26.02	4p15.2 → p14	13.37	NB	None given
44	4q31.21—> qter	35.3	4q31.21—> q32.1	11.49	34.8 years	Unspecified intellectual disabilities
45	5q31.3 → qter	40.83	5q31.2 → q31.3	0.906	1.5 years	None given
46	8pter → p12	31.31^a^	8p12 → p11.21	11.03	POC	Cystic hygroma
47	8pter → p21.1	24.38	8p21.2 → p12	5.2	25 years	Brain deformity, developmental delay, hearing loss
48	8q24.3 → qter	0.19	8q24.13- > q24.3	23.31	AF	Thickened NT, suspected heart defect, cleft palate
49	9pter → p22.3	15.47	9p22.3 → p21.3	9.66	5.9 years	Developmental delay
50	17pter → p13.3	1.91	17p13.3 → p13.2	3.78	13 years	Developmental delay
51	17q25.3 → qter	2.23	17q25.3 → 25.3	3.07	2.6 years	Delayed milestones
52	21q21.1 → qter (70%, 30% del)	28.1	21q11.2 → q21.1 (3.4 copy)	5	POC	Advanced maternal age
53	22q13.31 → qter	3.57	22q13.2 → q13.31	5.23	1.1 years	Microcephaly

^a^Mosaic deletion/duplication-triplication:arr 8p23.3p23.1(158048–31307174)x1[0.2], 8p23.1(31307174-42334760)x4[0.7]

This study offers evidence that segUPD has occurred secondarily to genomic corrections of deletions, derivative chromosomes, and terminal deletions contiguous with copy number gains. Specific examples of cases associated with various subcategories from the tables are highlighted as follows.

### SegUPD associated with corrections of derivative chromosomes

**Fig. 5:**
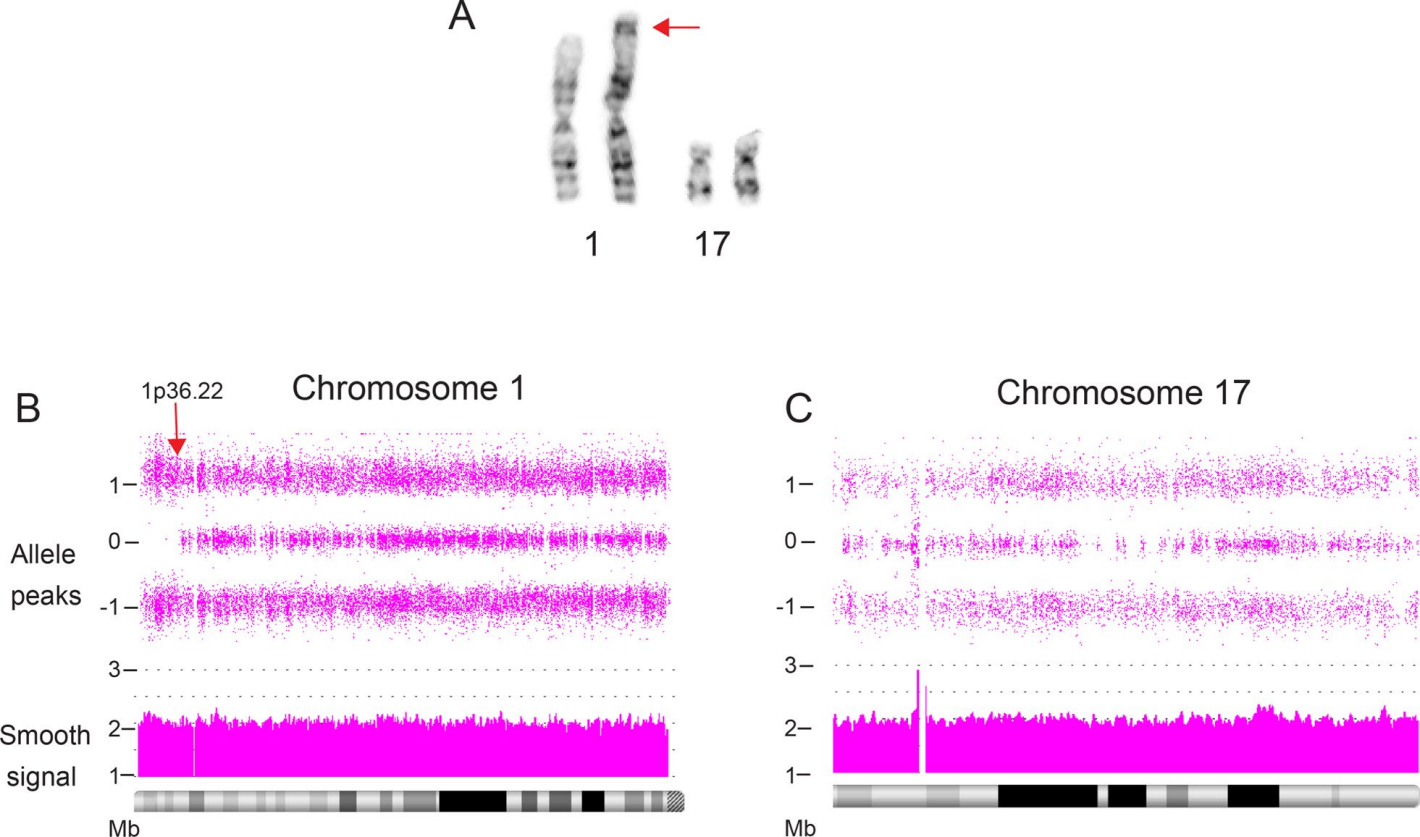
Correction of der(1)t(1;17) in case 1. **A** Partial karyotype of the 16 week amniocyte analysis in case 1. FISH and parental studies confirmed a de novo unbalanced derivative (1)t(1;17)(p36.3;q21) (arrow). **B**, **C** Blood array analysis at age 9 showed a terminal 9.4 Mb copy neutral ROH on chromosome 1 initiating at band p36.22 (arrow) with no deletion of 1p or evidence of partial trisomy 17

Case 1 illustrates homologous recombination-based correction of an unbalanced derivative chromosome 1 [der(1)t(1;17)(p36.3;q21)] that was detected in 48 of 50 amniocyte metaphases from a 21.4 week pregnancy (Fig. 5A). The indication was choroid plexus cysts and the pregnancy was continued to term. Nine years later, the proband had a peripheral blood microarray study, due to a clinical phenotype of an ependymal cyst, abnormal electroencephalogram (EEG), global developmental delay, hearing impairment, precocious puberty, enuresis, and severe intellectual deficit. The CMA showed a non-mosaic 9.39 Mb terminal ROH initiating at 1p36.22 (Fig. 5B), with no evidence of a derivative-related deletion of 1p or partial trisomy of 17p (Fig. 5C). A concurrent G-band blood analysis was normal, with no evidence of the der(1)t(1;17) in 105 cells. A subsequent buccal microarray analysis showed the same 9.39 Mb ROH on 1p, but again, no evidence of a residual derivative 1 imbalance. Parental chromosome analyses were normal and maternal SNP microarray comparisons were consistent with maternal segUPD in the ROH region. Thus, the results are consistent with a de novo paternal origin of the derivative chromosome 1 (Additional file 1: Figure S1).

**Fig. 6:**
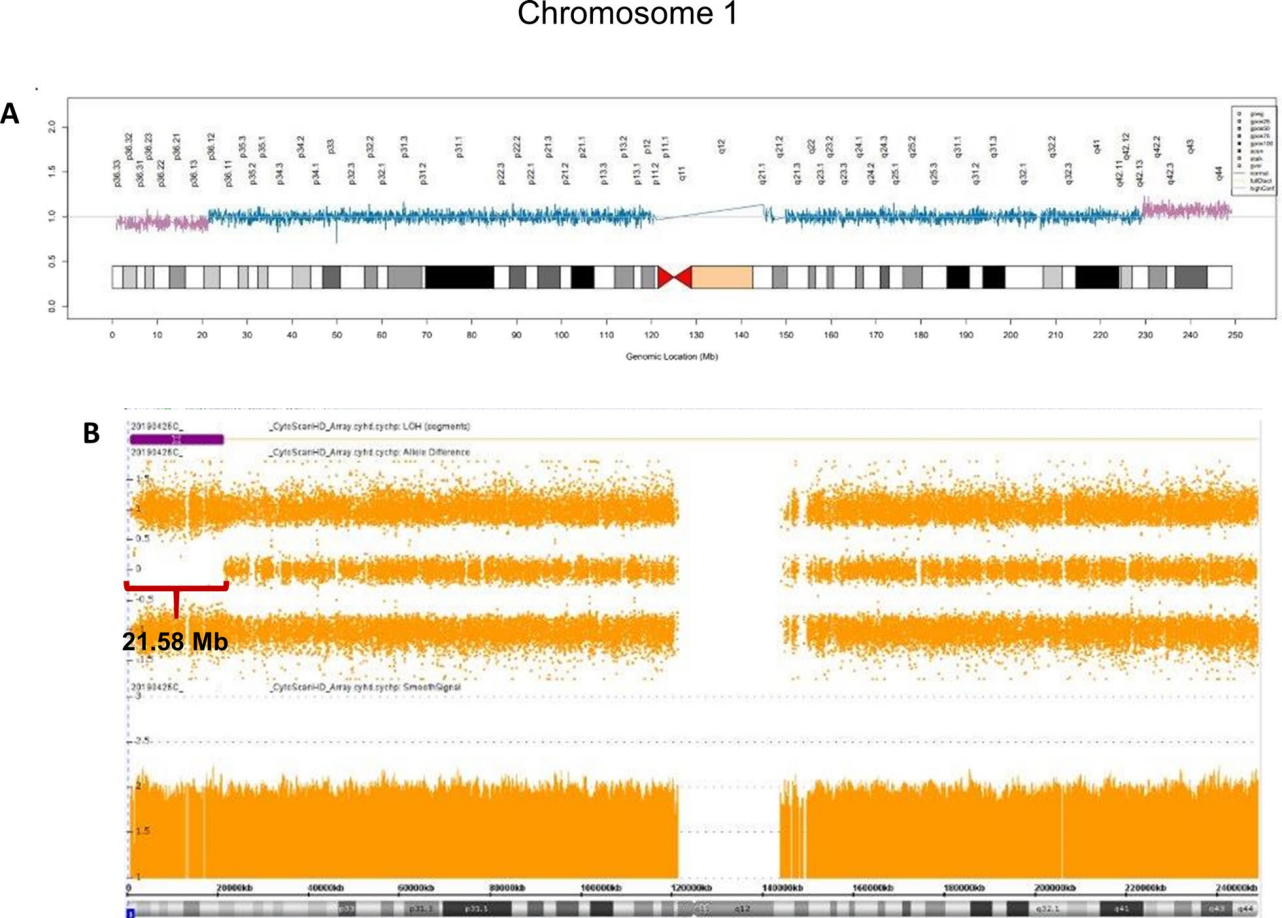
Possible inversion recombinant correction in case 3. **A** NIPT study, referred due to a cardiac lesion, showing a terminal deletion of 1p and a duplication of terminal 1q (**A**), with both equivalent to the 13% fetal fraction, consistent with non-mosaic fetal alterations. **B** Subsequent microarray analysis at 23 weeks of gestation showed no dosage changes of chromosome 1, although a 21.58 Mb terminal 1p ROH was present (**B**)

The noninvasive prenatal testing (NIPT) result of case 3 showed a terminal deletion of 1p and a duplication of terminal 1q equal to the fetal fraction at 14 weeks gestation, indicating an apparent non-mosaic fetal imbalance (Fig. 6A). This imbalance is consistent with the inheritance of a possible pericentric inversion recombinant of chromosome 1. A subsequent amniocyte CMA at 23 weeks showed no dosage changes of chromosome 1, although a 21.58 Mb terminal 1p ROH was present enabling interpretation of the terminal 1p ROH as apparent segUPD (Fig. 6B). Parental chromosome studies were recommended to rule out a recombinant chromosome from a balanced inversion carrier, but only maternal (normal) testing was available.

Another patient (case 5) was referred for late gestation NIPT (25 weeks), which showed an increased risk for a non-mosaic terminal 1p36 deletion and mosaic (37%) gain of terminal 18q. Post-delivery follow-up revealed the same non-mosaic 1p36 deletion, but no 18q duplication in a placental CMA analysis. A blood CMA analysis showed that a copy neutral ROH had replaced the deleted interval consistent with deletion repair occurring from the proximal end of the deletion.

**Fig. 7:**
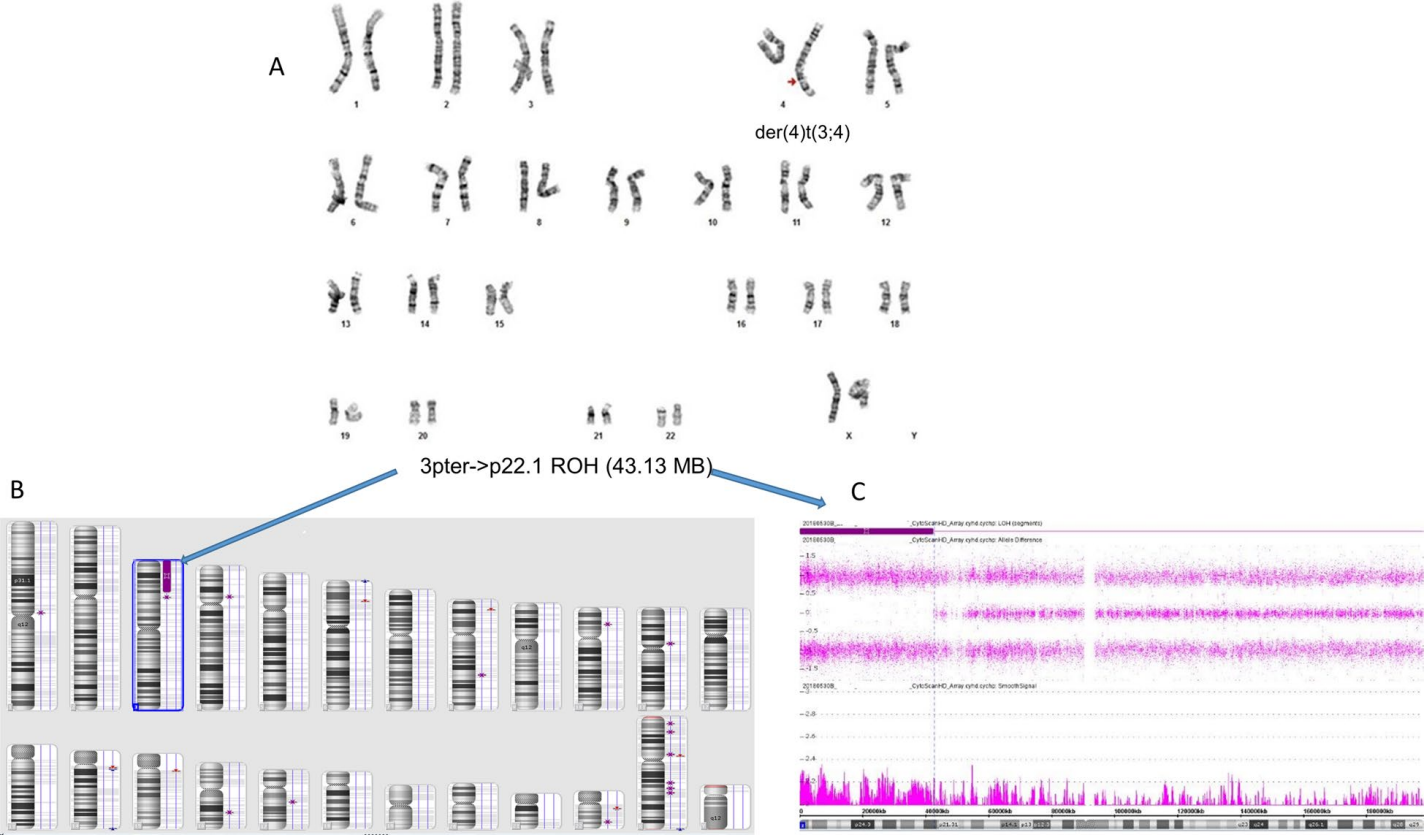
Novel correction of a derivative 4 in case 6. **A** Karyotype of the mosaic derivative(4)t(3;4)(p22;q35) still present in 5% of the patient lymphocytes as an adult. **B**. Whole genome view of ROH greater than 1 Mb from buccal cell CMA. **C** Isolated view of 3p terminal ROH. SegUPD confirmation is in Additional file 1: Figure S2

**Fig. 8:**
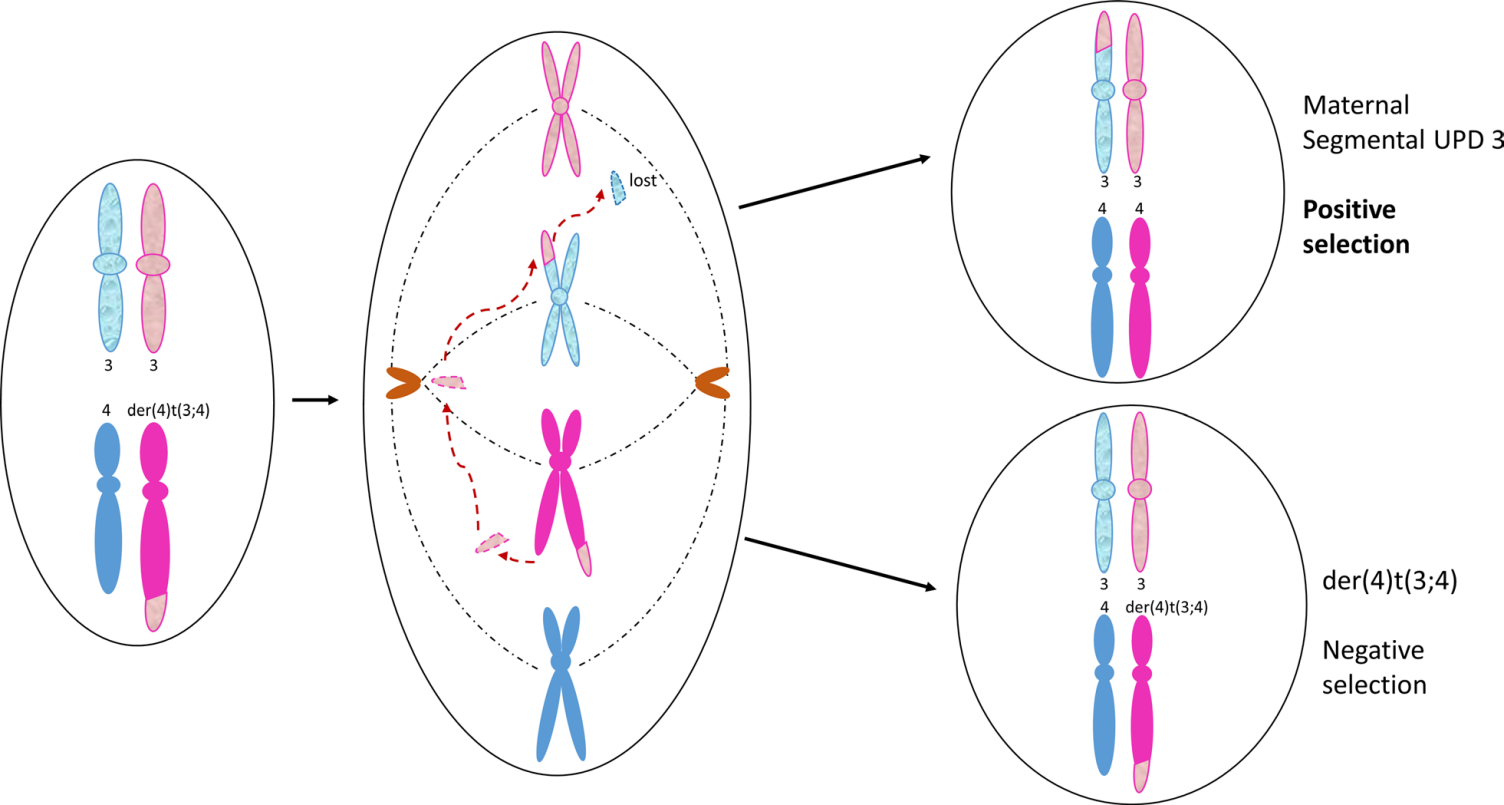
Apparent mechanism for segUPD 3p in case 6. Mitotic recombination at 3p22 and distal fission at 4q results in loss of the 3p segment from the derivative 4 and segUPD for 3p

A G-band chromosome analysis of a 4-year old female with developmental delay revealed mosaicism for an unbalanced translocation, der(4)t(3;4)(p22;q35), in 40% of the metaphases examined (Case 6, Fig. 7A). Twenty years later, the proband was referred for excessive weight gain and a clinical reevaluation. A blood chromosome reanalysis showed the unbalanced derivative in 5% (6/120) of lymphoid metaphases, while the CMA showed ~ 8% partial trisomy (3pter → p22.1) with complete homozygosity of the same 43.13 Mb region and no alterations of 4q (Fig. 7B, C). A subsequent buccal microarray showed normal dosage and the same 3p ROH. A maternal-proband CMA allele comparison and normal maternal blood karyotype was consistent with maternal segmental UPD (segUPDmat) and de novo origin of the unbalanced translocation (Additional file 1: Figure S2). A diagram of the apparent mechanism is shown in Fig. 8.

**Fig. 9:**
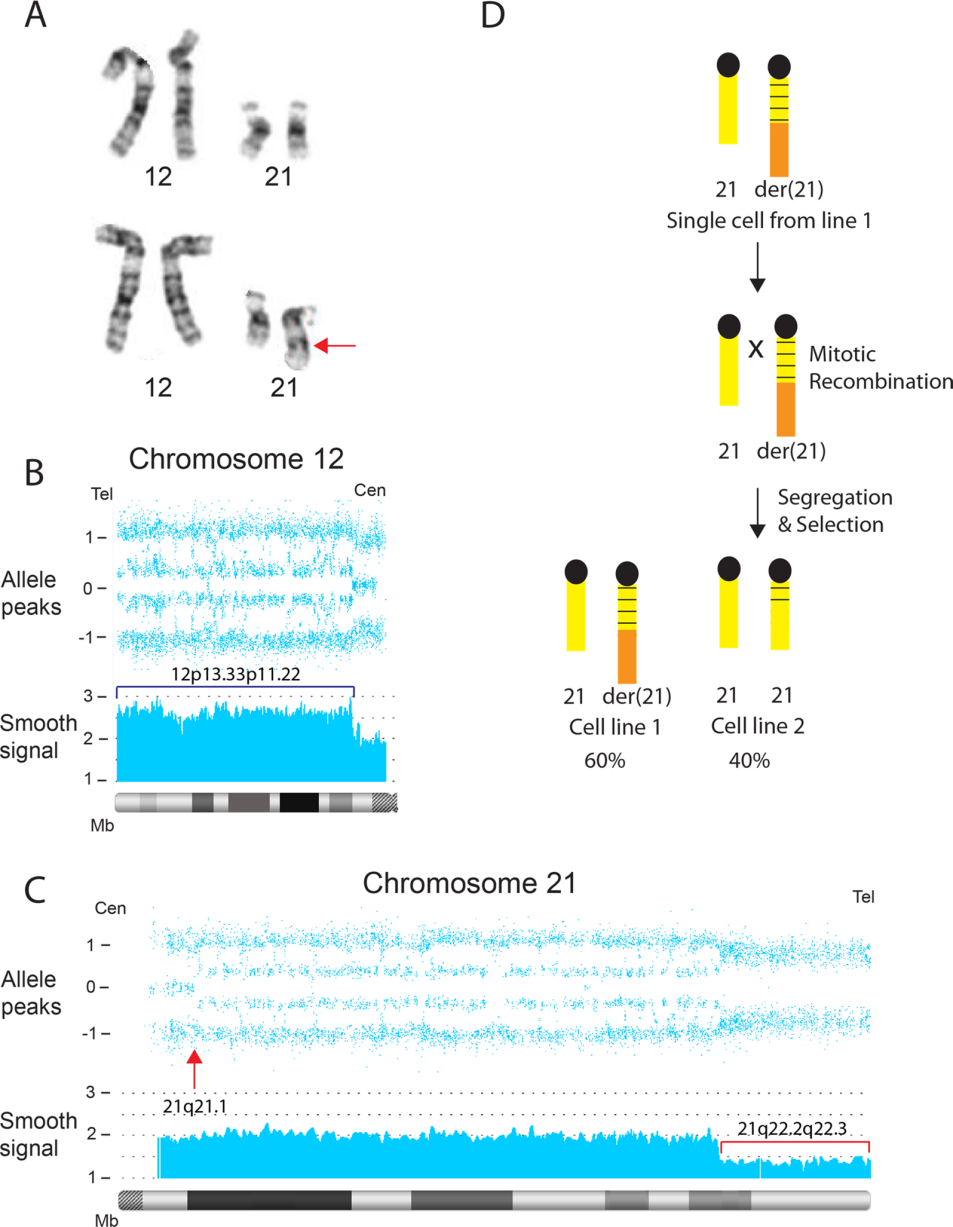
Incomplete correction of the translocation derivative 21 in case 35. **A** Partial karyotype showing mosaic cell lines in case 35. Normal cell line (top panel) and derivative (21)t(12;21)(p11.22;q22.2) cell line (bottom panel, arrow). **B** CMA of chromosome 12 shows a mosaic terminal duplication (bracket) of 30.51 Mb in ~ 60% of cells. **C** CMA of chromosome 21 shows a mosaic terminal deletion (smooth signal track, bracket) of 7.12 in ~ 60% of cells. Arrow shows the initiation site of mitotic recombination at 21q21.1, resulting in replacement of the der(21) with a segment from the normal homologue and the obligate homozygotic allele dosage. **D** A single cell in which the der(21) initiates mitotic recombination with the normal homologue. Segregation and selection results in a second cell line with a normal copy number for chromosome 12 and 21, resulting in segUPD 21q21.1->qter

Peripheral blood from a 29-year old male referred for a developmental disorder was tested using microarray and cytogenetic analyses (Case 35). Chromosome studies revealed mosaicism for two cell lines: ten cells showed a derivative chromosome 21 and the remaining ten cells showed a normal 46, XY karyotype (Fig. 9A). Concurrent microarray studies revealed a ~ 60% mosaic 7.12 Mb terminal deletion of chromosome 21(q22.2 → qter) and a ~ 60% mosaic 30.51 Mb terminal duplication of chromosome 12(pter → p11.22) (Fig. 9B, C). The CMA also showed a 24.38 Mb mosaic copy- neutral ROH in chromosome 21(q21.1 → q22.2), proximal to the mosaic deletion (Fig. 9C) with ~ 40% homozygosity. The mosaic terminal deletion showed no evidence of heterozygous alleles, consistent with a prezygotic origin, while the proximally adjacent segment showed heterozygous alleles, consistent with a postzygotic origin. The proposed mechanism for this result is mitotic recombination that occurred at 21q21.1 (Fig. 9C, arrow), proximal to the rearrangement breakpoint, resulted in replacement of most of the derivative chromosome 21 with a segment from the normal homologue (Fig. 9D).

### SegUPD associated with corrections of deletions

**Fig. 10:**
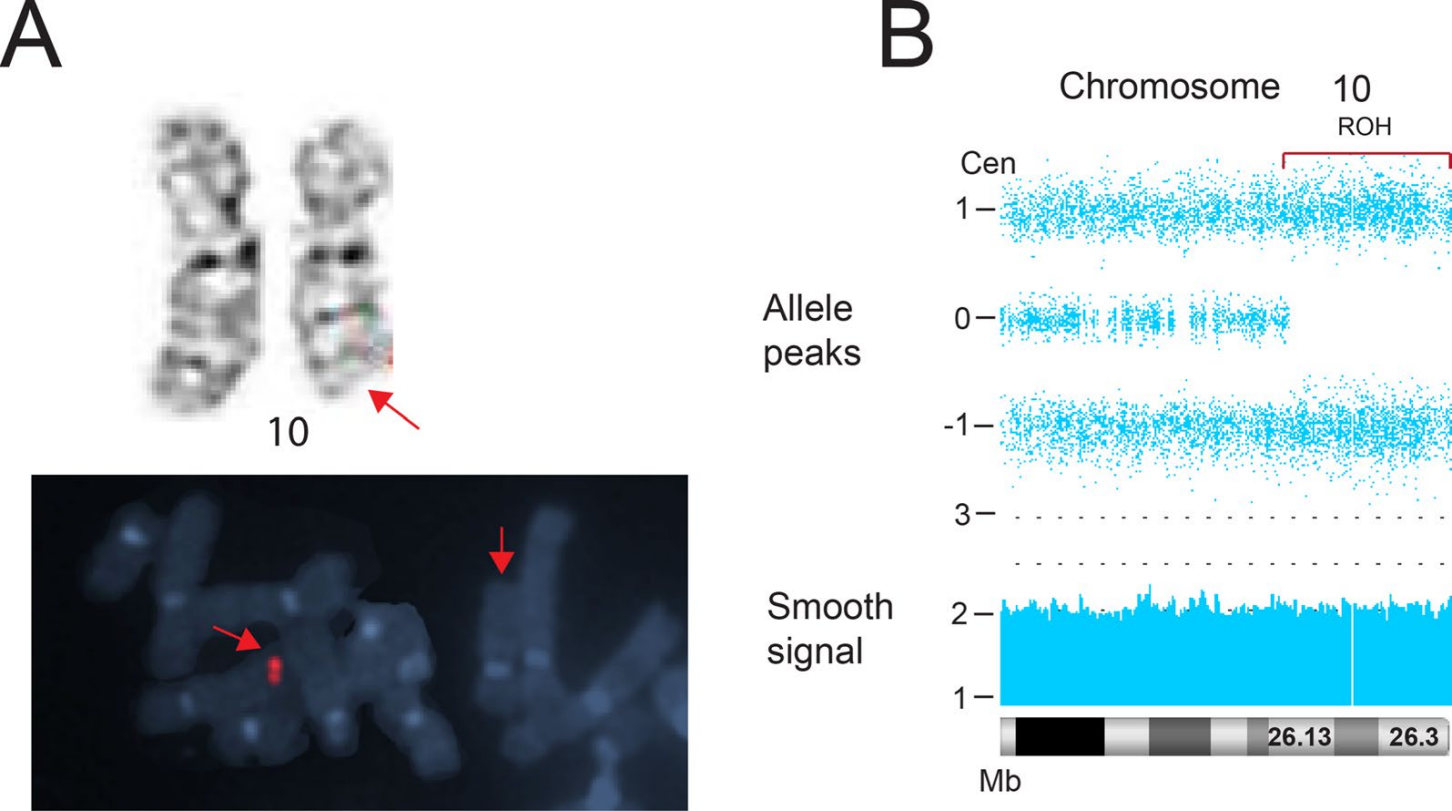
Analysis of prenatal deletion in case 9. **A** Case 9 showing a deletion of 10q present in all cells from a CVS chromosome analysis (top panel, arrow) that was confirmed by a region-specific FISH probe (bottom panel, arrow). **B** Post-delivery blood CMA revealing a copy neutral terminal ROH on chromosome 10 initiating at band q26.13 (bracket). Confirmation of segUPD is shown in Additional file 1: Figure S3

The chorionic villus sample (CVS) analysis in case 9 showed a G-band visible and FISH-confirmed terminal deletion of the long arm of chromosome 10, initiating at band q26.1 in all cells (Fig. 10A). In the absence of ultrasound abnormalities, the parents elected to continue the pregnancy. A post-delivery blood CMA revealed a non-mosaic copy-neutral terminal ROH of 11.12 Mb on chromosome 10 (q26.13 → qter), corresponding to the former deletion interval (Fig. 10B). Parental CMA allelic comparisons were consistent with maternal segUPD (Additional file 1: Figure S3). The newborn appeared clinically normal and, at the age of 3, there appeared to be no deletion-related symptoms.

**Fig. 11:**
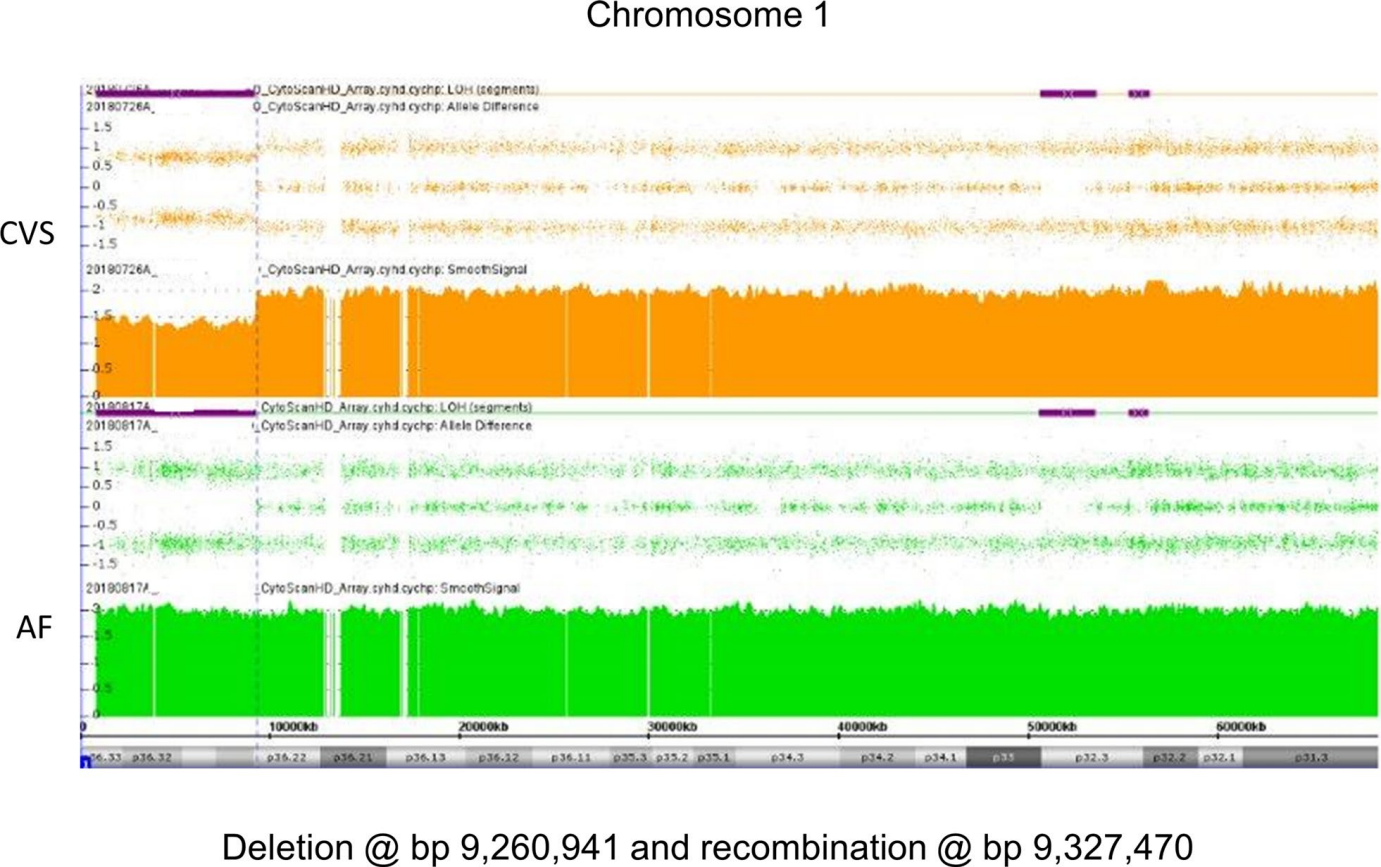
CVS and AF CMAs in case 2. 1p36.22 deletion in CVS analysis (top panel) with a correction to apparent segUPD in the AF analysis (bottom panel). The close proximity of the AF ROH correction initiation site to the original deletion site is consistent with a recombination event in close proximity to the deletion breakpoint

An NIPT study consistent with a terminal 1p deletion led to a CVS CMA in case 2. The CMA showed a mosaic deletion/ROH without heterozygous alleles at terminal 1p and a subsequent amniocyte CMA revealed a copy-neutral ROH in the deletion interval, consistent with a germline error correction and segUPD (Fig. 11). The presence of normal cells (40%) in the CVS indicate that correction initiated before fetal development and normal fetal ultrasound results suggested the correction may have preceded clinical effects.

**Fig. 12:**
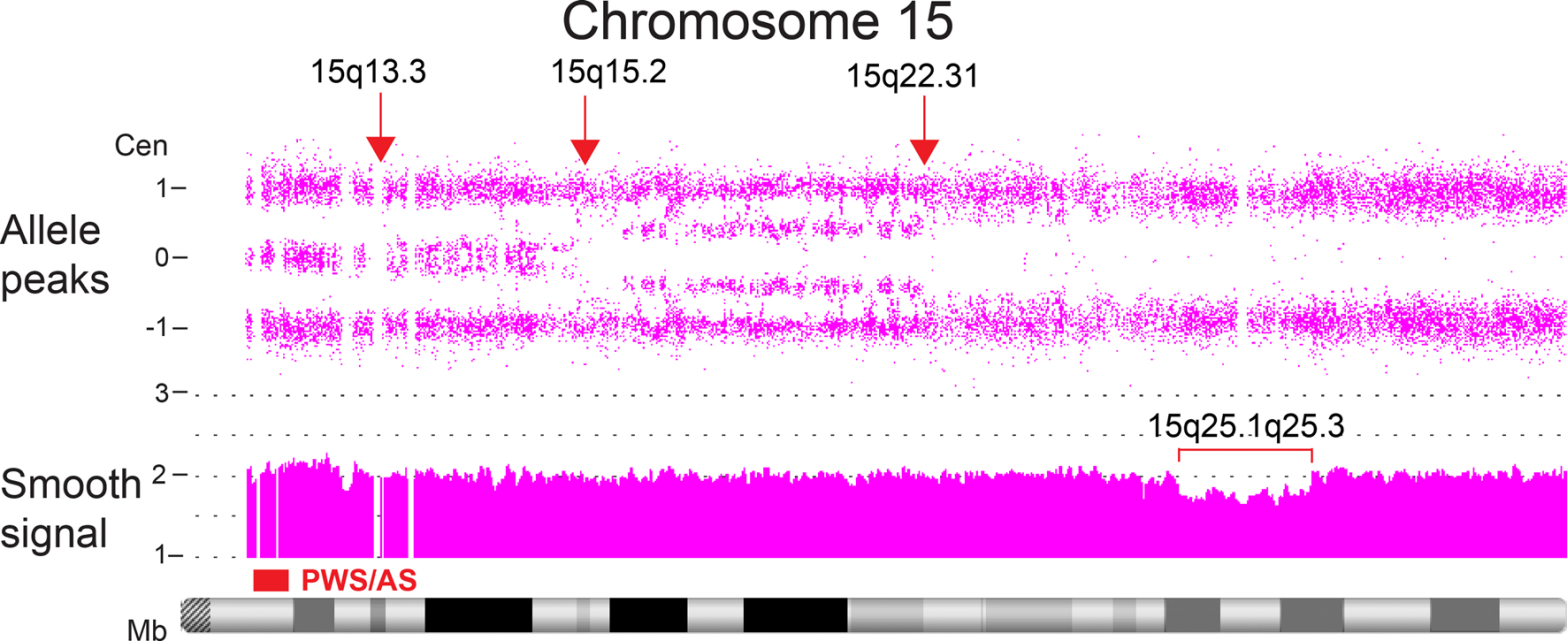
Mosaic segUPD15 associated with 3 distinct corrections of an interstitial deletion detected in case 30. Three deletion repair cell lines initiate at different mitotic recombination sites (arrows). The deletion (bracket) is still present in ~ 25% of cells. Note that the allele difference tract shows ~ 75% of cells with homozygosity in the location of the deletion, consistent with the percentage of deletion correction

Microarray peripheral blood studies of case 30, a 12-year old female referred for developmental delay and short stature, revealed an 8 Mb mosaic (~ 25%) interstitial deletion of chromosome 15 (q25.1q25.3), along with three different percentages of mosaic homozygosity associated with three proximal recombination sites (Fig. 12). The most proximal region extended from 15q13.3 → q15.2, with an allele difference tract consistent with 15% homozygosity. The next region extended from 15q15.2 → q22.31, with the tract consistent with ~ 46% homozygosity, and the most distal region extended from 15q22.31 to the terminus of chromosome 15 with ~ 70% homozygosity.

### SegUPD with a possible postzygotic origin of allelic imbalance

Three cases suggest a somatic origin of allelic imbalance, resulting in apparent segUPD. In case 8, the original CVS CMA showed 50% copy-neutral homozygosity at terminal 7q and the subsequent amniotic fluid CMA was 100% homozygous in the same region. The placental absence of a dosage deficit in the presence of a terminal mosaic ROH on chromosome 7 is not consistent with deletion rescue; the further homozygous evolution in the amniocyte analysis is suggestive of active selection for the gene converted segment.

**Fig. 13:**
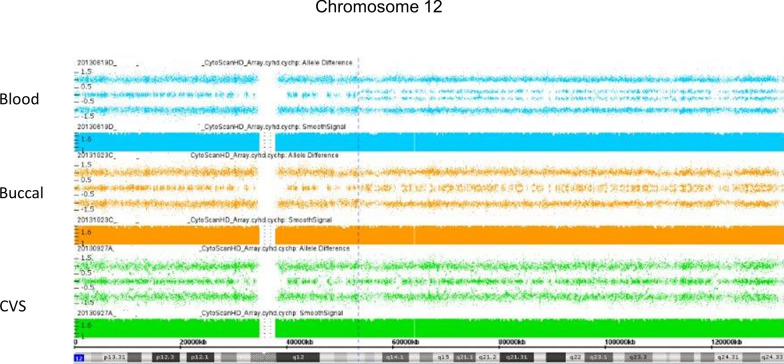
Evidence for somatic origin of mosaic segUPD 12 in case 24. Mosaic apparent segUPD12 detected in the blood of a newborn (top) and, to a lesser extent, in a buccal swab (middle), but absent in the placenta (bottom) in case 24

The results of 3 CMAs of a newborn male from case 24 showed a normal placental analysis, but mosaicism for a 12q terminal ROH and apparent segUPD in both blood and buccal cell CMAs (Fig. 13). The patient phenotype could not be specifically linked to either partial monosomy or to a mutation in the segUPD interval. An unrelated pathogenic mutation subsequently detected in the *ZEB2* gene (2q22.3) was consistent with the patient symptoms (Mowat–Wilson syndrome, OMIM# 235730).

Case 34, a 35-year old male, showed progeria-like symptoms that progressed with age. The CMA from peripheral lymphocytes demonstrated two apparent segUPD subpopulations with separate mitotic recombination initiation sites on the long arm of chromosome 19.

### SegUPD associated with contiguous duplication and terminal deletion

**Fig. 14:**
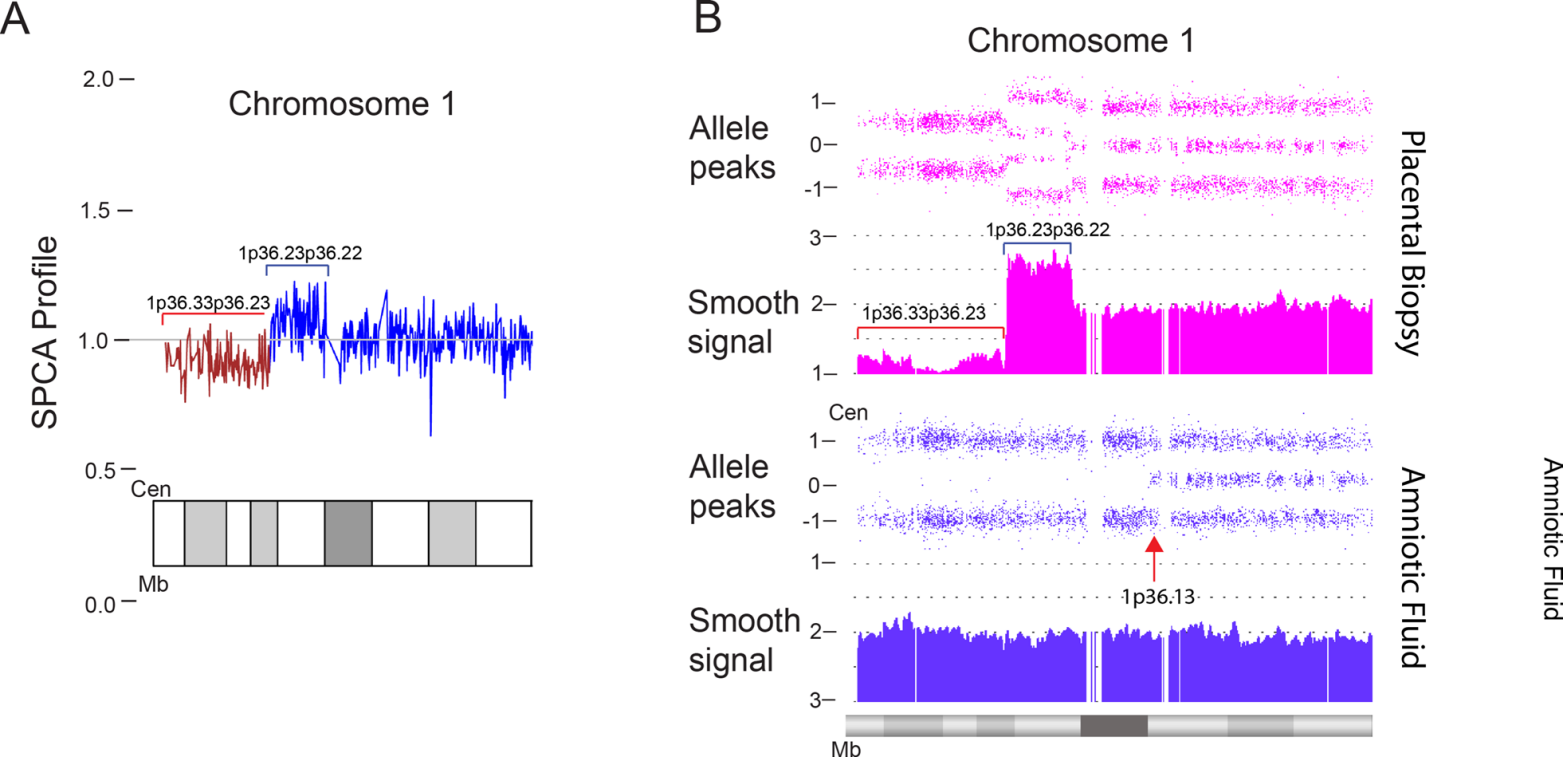
Temporal prenatal analyses in case 4. **A** NIPT analysis showing an 8.72 Mb terminal deletion (red bracket) contiguous with a 3.44 Mb duplication that extends to 1p36.22 (blue bracket). **B** Amniocyte CMA revealing a terminal 16.32 Mb copy neutral ROH initiating at band 1p36.13 (lower panel). Pooled placental analysis (top panel) showing ~ 80% mosaicism for the alterations initiating at 1p36.22. Note that the ROH initiates proximally to the duplication

DNA isolated from routine placental-based NIPT screening studies from a 16-week female fetus showed an increased risk for an ~ 8 Mb terminal deletion of 1p with a contiguous proximal duplication (case 4, Fig. 14A). A subsequent amniocyte microarray analysis revealed a normal female dosage with a terminal 16.32 Mb ROH on the short arm of chromosome 1 that extended proximally to 1p36.13 (Fig. 14B). This ROH was shown to be segUPD of maternal origin by parental microsatellite analysis. At delivery, only normal G-banded metaphases from lymphocytes were detected in 50 cells analyzed. A microarray from pooled placental biopsies post-delivery showed equivalent 80% mosaicism for an 8.72 Mb terminal deletion and a contiguous 3.44 Mb duplication extending from 1p36.23 to 1p36.22, in agreement with NIPT studies. The ROH, thus, originated 4.14 Mb proximal to the duplication, consistent with homologous recombination-mediated repair occurring at a location distant from the rearrangement.

### SegUPD associated with a contiguous triplication

**Fig. 15:**
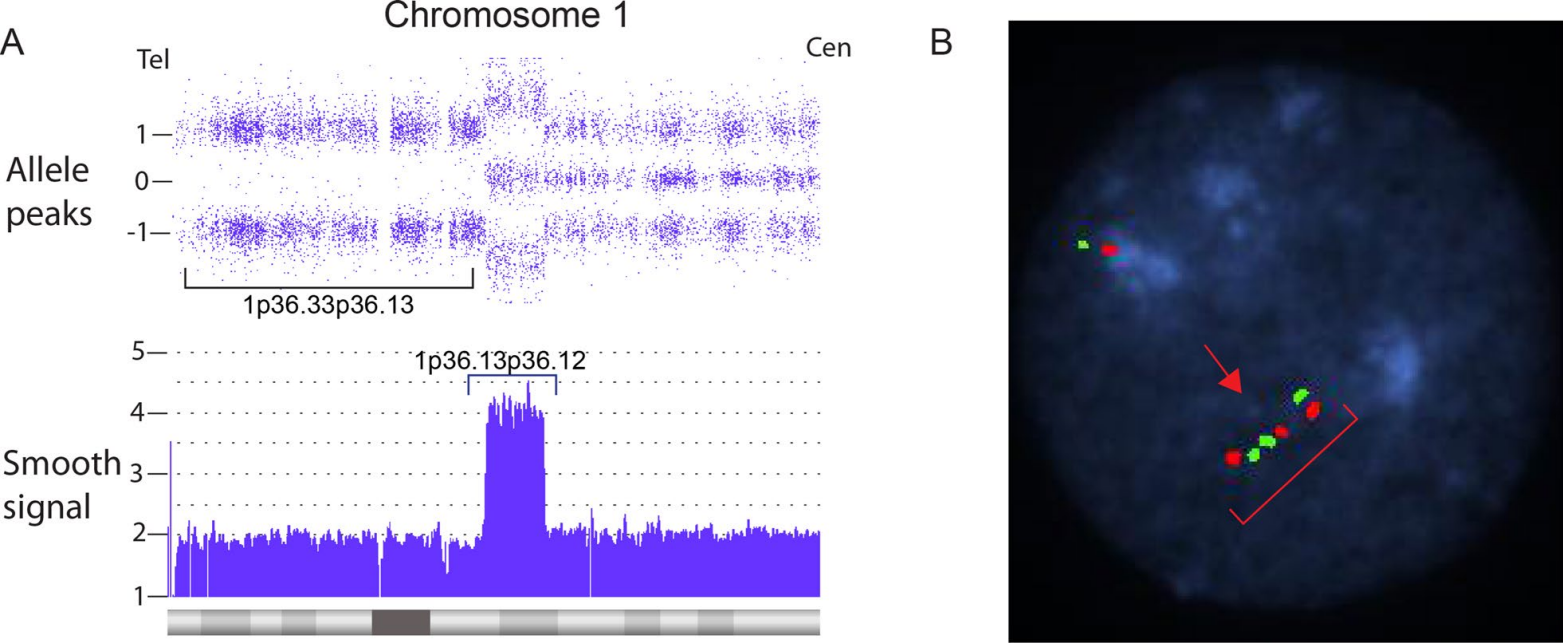
CMA and FISH analyses of a case of segUPD associated with a triplication. **A** Microarray image of case 38, shows 3.56 Mb triplication (blue bracket) of bands p36.13p36.12 on chromosome 1 with an exclusive 2:2 (AABB) heterozygote allele pattern and a contiguous copy neutral terminal 19.35 Mb ROH (bracket). **B** A dual target interphase FISH image showing triplication of 1p (bracket) with inverted orientation of the middle segment (arrow)

**Fig. 16:**
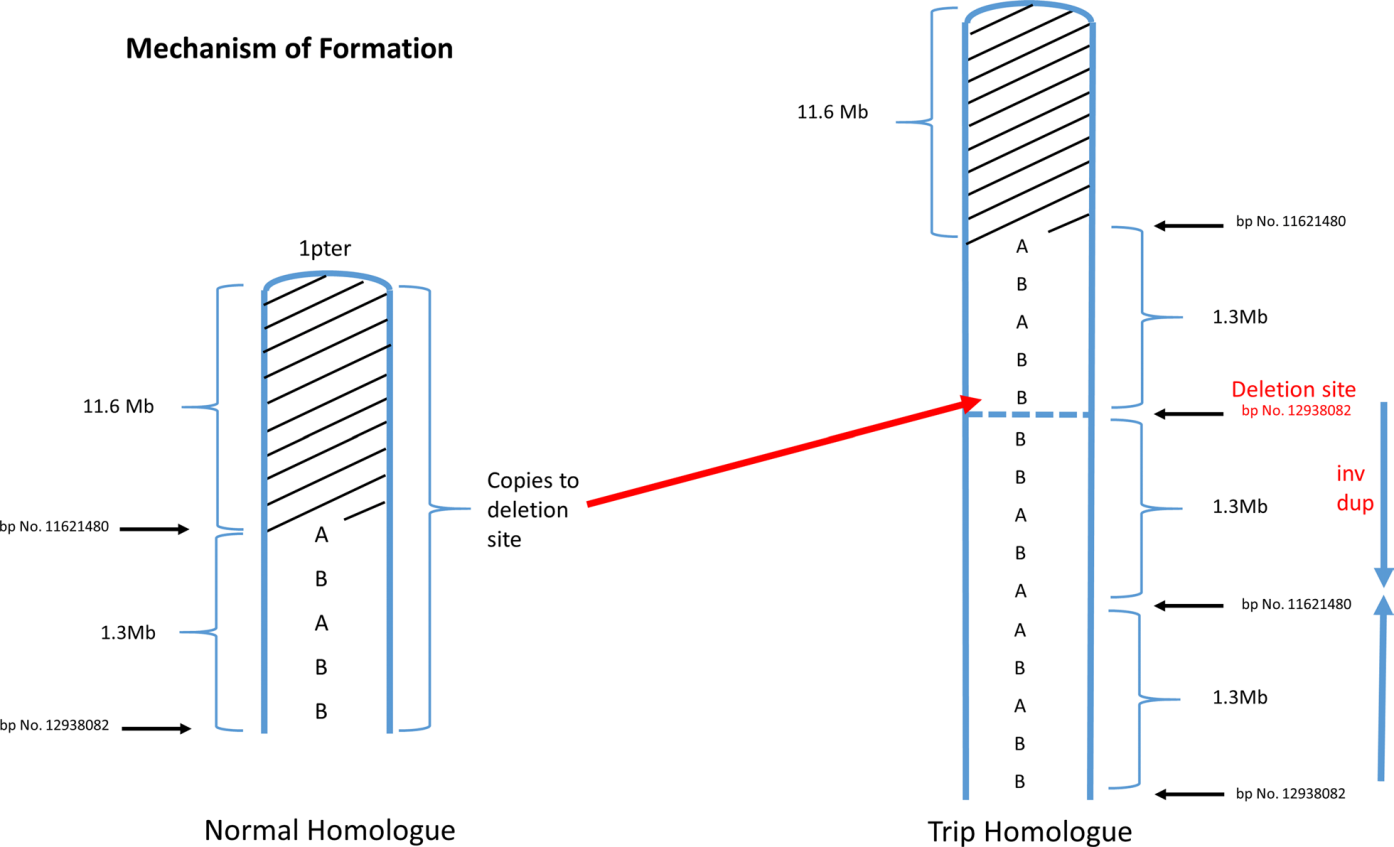
Postulated mechanism of segUPD associated with triplications. After passage of a meiotic derived inverted duplication with a contiguous terminal deletion to the zygote, there is somatic rescue by the normal homologue, initiating from the most distal sequence of the inverted duplication. This results in adding a third copy to the duplication and exclusive AABB heterozygote alleles in the CMA (rather than three or four tracts), while correcting the deletion imbalance with terminal segUPD

Terminal segUPD with a contiguous triplicated interval was found in 17 cases (Table 3) and is exemplified by case 38, a 7.6-year old male. The blood leukocyte CMA showed a 19.35 Mb terminal 1p ROH with a proximal contiguous 3.56 Mb triplication extending from 1p36.13 to 1p36.12 (Fig. 15A). A defining feature of cases in this group is an exclusive 2:2 heterozygous allele dosage (AABB) in the triplicated segment with no 3:1 relative allele dosage [20] (Fig. 3) and a tandem triplication structure in a single homologue with an inverted middle copy, as confirmed by interphase FISH (Fig. 15B). A mechanistic ideogram of this class of rearrangement is presented in Fig. 16, which shows replacement of a terminal deletion and adjacent proximal inverted duplication with a terminal region of segUPD and adjacent triplication by somatic telomere capture from the normal homologue.

**Fig. 17:**
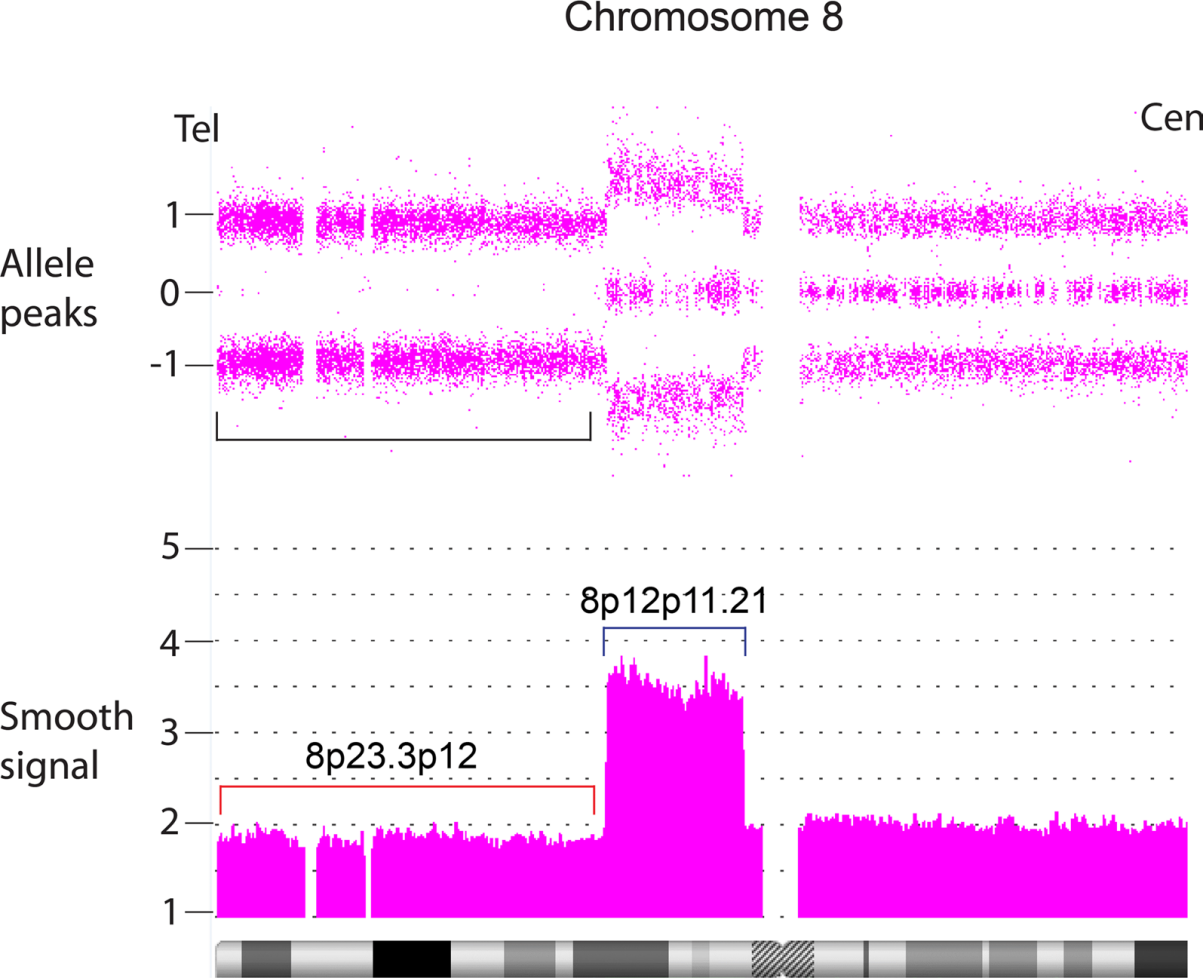
Mosaic example of a segUPD/triplication correction. The fetal demise from patient 46 showed terminal homozygotic alleles and smooth signal dosage consistent with ~ 80% replacement of a terminal 8p deletion/proximal duplication with a copy of the terminal segment from the normal homologue, resulting in terminal segUPD visualized by a terminal ROH (bracket) and a contiguous triplication with the characteristic exclusive balanced AABB heterozygote alleles (smooth signal track, blue bracket). Replacement of the deletion with material from the normal homologue appears to have initiated at the distal end of the contiguous duplication

A CMA from placental analysis of a fetal loss provides an example of an apparent incomplete correction of an 8p alteration in this subgroup (case 46, Fig. 17). A 31.31 Mb terminal short arm ROH showed a 20% dosage deficit. Contiguous with the deletion was an 11 Mb copy gain extending from band p12 to p11.21, which displayed the characteristic equal 2:2 dosage of heterozygous alleles in this subgroup with copy number of approximately 3.7. The dosages are commensurate with an ~ 80% somatic correction of the original deletion and gain of an extra copy of the duplicated region.

### Studies of gonadal mosaicism

**Fig. 18:**
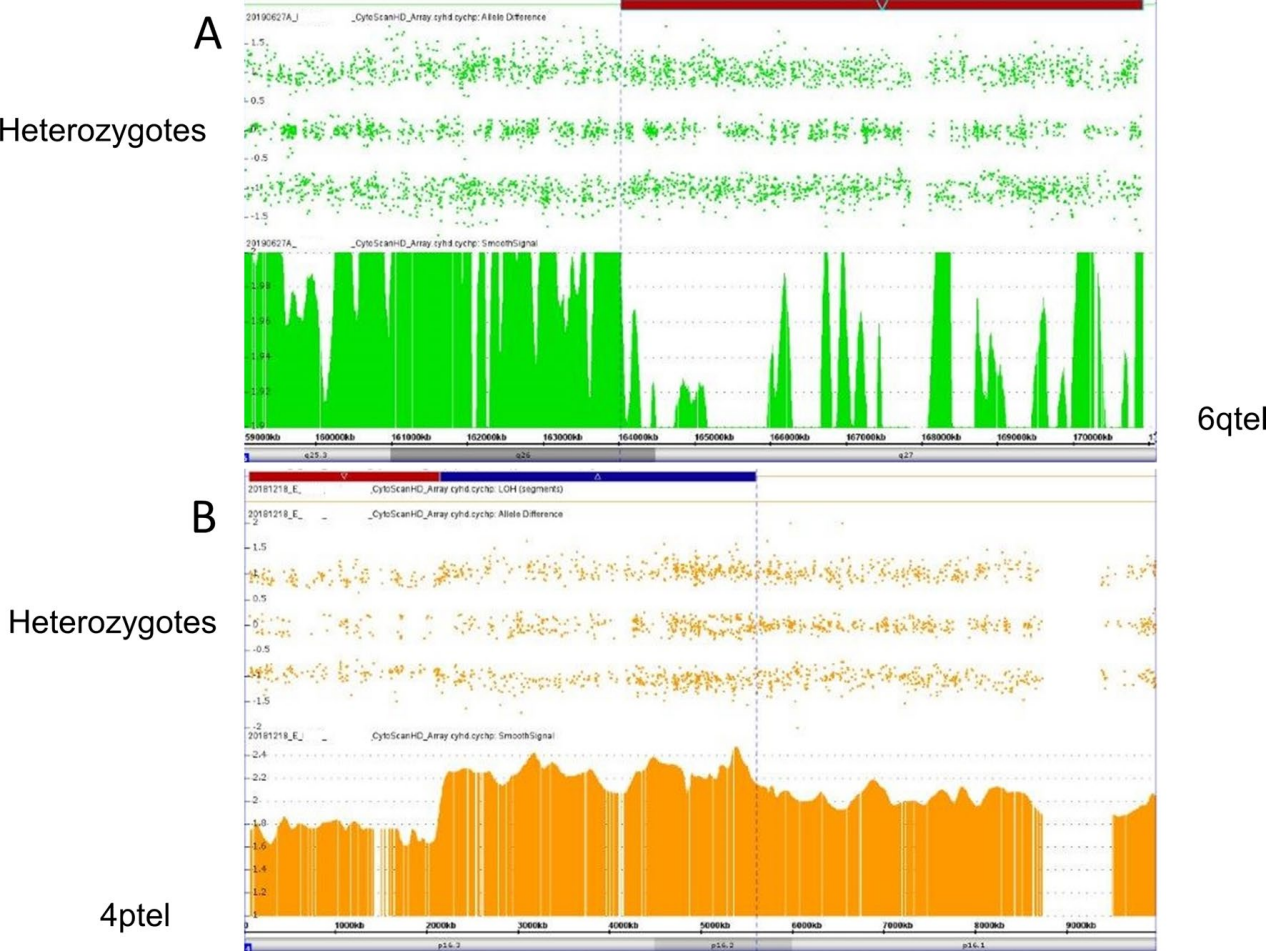
Study showing post zygotic origin of gonadal mosaicism. **A** Maternal buccal CMA follow-up from the mother of two 6q- offspring revealing a mosaic ~ 11% deletion, red bar. **B** Maternal buccal follow-up from the mother of two offspring with a 4p deletion-duplication showing ~ 25% deletion (red bar) and duplication (blue bar). Note that the presence of heterozygote alleles in the allele difference tract in the deletion interval are consistent with a post zygotic origin of the gonadal mosaicism and inconsistent with segUPD related correction

Two maternal CMAs (Fig. 18) were pursued to determine the etiology of apparent gonadal mosaicism following repeat alterations, a terminal deletion contiguous duplication of 4p and deletion of 6q, in two pregnancies of clinically normal mothers. In both cases, a previous child was born with identical alterations (terminal deletion/proximal duplication of 4p and terminal deletion of 6q, respectively). In both families, the children were severely affected, while the phenotypically normal mothers showed mosaicism for the alterations in buccal cells (25% and 11%, respectively). The mother of the 6q- children also showed the deletion in blood cells (35%). These alterations were apparently somatically-derived and not associated with segUPD. However, they are included to show that allele dosage patterns in CMAs can differentiate corrections of somatically-derived alterations, in which heterozygous alleles are present in the deletion interval, from meiotic alterations, which are devoid of heterozygous alleles and associated with segUPD. Heterozygous alleles can be present in mosaic deleted intervals only when the normal cells demonstrate biparental inheritance in the deletion interval and, thus, heterozygosity indicates that the deletion arose post-zygotically. Conversely, the template copy generated from the undeleted homologue in mitotic recombination-based correction of meiotic errors has no heterozygote alleles (also see Fig. 9C from patient 35 above).

A possible case of gonadal mosaicism due to incomplete mitotic correction of a meiotic imbalance is provided by case 12, a blood CMA from a 42-year old, apparently clinically normal female with a history of 5 fetal losses. The array showed a long terminal ROH on chromosome 15q, suggestive of UPD15. Since the ROH was distal to the Prader-Willi/Angelman Syndrome region and the patient did not have either imprinting syndrome, whole chromosome UPD was unlikely. Paternal segUPD15 was confirmed (Additional file 1: Figure S4). Because oocytes are produced early in embryogenesis with no mitotic opportunity to correct, the patient’s infertility may be associated with a high proportion of oocytes carrying a deletion or some other aberration.

## Discussion

### General mechanism: SegUPD and selection

Most of the patient studies in this report demonstrate segUPD that correlates with mitotic recombination-based somatic correction of apparent meiotic imbalance involving terminal chromosome arms. Mosaic seqUPD may also arise from a normal zygote related to an expansion of a clonal population of cells derived from a single progenitor cell. These cells show no copy number alteration, but demonstrate selection driven by homozygosity of a gene in one of the homologues providing proliferative advantage, as is the case in Beckwith–Wiedemann syndrome. Both mechanisms appear to require that stochastic double-stranded breaks occur, followed by inter-homologue sister chromatid exchanges initiating this process [21], after which there is segregation of somatic recombinants and subsequent proliferation of those recombinants with a selective advantage (Fig. 2).

Patent evidence of a meiotically-derived terminal deletion is depicted in case 9, which shows a 10q terminal deletion in all cells of a CVS chromosome analysis and complete correction in a subsequent newborn blood CMA. Evidence of frequent placentally-confined aneusomy supports the assertion that selection against a genomically unbalanced cell line is much greater in the fetus than in the placenta [2, 22]. The placental-fetal stage at which the imbalance completely corrects is likely to be critical in regard to potential developmental effects.

Four NIPT studies that preceded CMAs, each involving terminal deletions of 1p, offer etiological insight.

The proximal loci of the original deletion breakpoint and the subsequent ROH appear to be the same in cases 2 and 3, consistent with the breaksite being the initiation site of mitotic recombination. The presence of normal cells in the CVS detected from case 2 suggests that correction initiated before fetal development, resulting in no fetal monitoring anomalies detected. In contrast, in case 3 neither the imbalance detected by NIPT nor the corrected amniocyte microarray showed evidence of mosaicism, so the timing of correction in the pregnancy and potential clinical effects are unclear. However, the fetal heart defect, which has been noted in some cases of distal 1p deletions, suggests that the correction occurred too late to prevent pathogenicity.

The NIPT correlative case 4 revealed correction of another common type of germ line alteration, terminal deletion/contiguous duplication. The early gestational NIPT dosage was consistent with this alteration at the 1p terminus, (confirmed by a post-delivery placental biopsy CMA), and the subsequent amniotic fluid CMA showed complete dosage correction. In contrast to cases 2 and 3, mitotic recombination initiated proximally to the rearrangement by breaksites established from the placental biopsy, which showed mosaicism for the rearrangements and, thus, was in the process of correction. Since dosage can be normalized by recombination at any point proximal to the imbalance, including the most proximal point of the imbalance, there would appear to be no specific selective advantage associated with the ultimate recombination site. Indeed, the random nature of proximal arm recombination initiation sites and the relative lack of interstitial sites in gene conversions has been noted in previous research [23–25].

The fourth NIPT case had a non-mosaic terminal 1p36 deletion and mosaic terminal 18q gain that was corrected in blood, but not in the placenta, consistent with two different somatic mechanisms. The absence of the 18q gain in the blood CMA strongly suggests that it was acquired in a population of placental cells because of the need for a 1p telomere via telomere capture [26]. However, there was subsequent selection for a euploid cell line with segUPD-related correction, rather than the telomere capture cell line with the deletion and partial trisomy. Neonatal symptoms of the 1p36 deletion syndrome indicate the correction was too late to prevent clinical effects.

The blood and buccal CMA analyses in case 1 show complete correction of a single derivative 1 of a t(1;17)(p36;q21), identified 9 years prior in amniocyte metaphases (Fig. 5). Unlike cases 4 and 9, the clinical effects in this child were clearly due to fetal involvement of the imbalance, since the der(1) was detected in 96% of the amniocytes. Fetal involvement of the aberrant cell line is unclear in cases 4 and 9, in which abnormalities were ascertained because of NIPT and CVS chromosome studies, respectively. This illustrates the difficulty in predicting clinical effects associated with a transient genomic imbalance due to the timing of correction in some prenatal studies.

Case 35 illustrates apparent incomplete correction of a translocation derivative chromosome [der(21)t(12;21)], which showed mosaicism for the aneuploid and diploid cell lines in G-banded lymphocytes. This illustrates the potential for a mosaic unbalanced translocation derivative to have been inherited, with the normal cells acquired somatically.

Case 6, the correction of a de novo der(4)t(3;4)(p22;q35)mat through apparent mitotic recombination with the paternal chromosome 3 homologue, is unique for a number of reasons. This was the only correction found that was mediated by a break in a chromosome other than in the original derivative. It was also the only case with a correction associated with a partial gain and no apparent loss. The complete correction in the buccal CMA and incomplete correction in the blood CMA would appear to be atypical, assuming that more rapidly dividing cells will selectively correct faster than slower dividing counterparts [27]. However, tissue specific selection presumably varies during differentiation, which can alter relative cell line involvement as found in Pallister–Killian syndrome [28].

Two cases with apparent maternal gonadal mosaicism displayed post-zygotically derived imbalances of 4p and 6q that were detected in blood and buccal cells. It may seem unlikely that an abnormal cell population with a post-zygotic origin would be sustained through development when competing with a normal cell population, but evidence of this occurring is provided by these familial studies. The reproductive recurrences of the 4p and 6q lesions were consistent with gonadal mosaicism due to an early developmental error that included the germ cells. Interestingly, the 6q- cells were found in maternal lymphocytes, while the 4p imbalance was normal in a blood CMA and mosaic in a buccal CMA. Again, this may relate to differences that can exist in tissue-specific selection.

A reproductive recurrence risk in a clinically normal individual who underwent genomic correction of a meiotic derived imbalance in early development is also possible, as revealed by case 12 (with a history of five fetal losses and paternal segUPD15). The patient’s infertility could be associated with a high proportion of oocytes carrying a deletion or some other aberration that failed to be corrected in those cells because of the timing of oocyte production in embryogenesis.

There may be a significant reproductive recurrence risk in an individual with segUPD. There are multiple studies supporting this possibility, with 3 of these supporting a possible “hot-spot” involving terminal 11q Jacobsen syndrome (JS) related deletions [29–31].

Multiple studies offer compelling corroborative evidence for both a high incidence of 11q deletion rescue and associated germ cell mosaicism [29–31]. Johnson, et al. report two fraternal brothers with the same JS-related deletion detected, although one was mosaic for the deletion. The maternal blood SNP microarray analysis revealed a copy-neutral, non-mosaic ROH, coinciding with the deleted region in her sons. SegUPD11 was confirmed in the mother and an additional maternal fibroblast CMA revealed mosaicism both for the deletion and segUPD. Not surprisingly, mild JS symptoms were present in the maternal clinical evaluation. Thus, this study suggests a strong predilection to 11q deletion correction, since both mother and son had 11q correction with clinical variability. In addition, the study illustrates variable tissue-specific selection and confirms that the correction process may not include oocytes. Gonadal mosaicism occurs either because of uncorrected gametes from meiotic lesions or from gametes with somatically derived lesions, differentiable based on the presence/absence of heterozygote alleles in parental testing.

### SegUPD associated with triplication

A distinct subset of 17 cases showed terminal segUPD located contiguous to triplications (Table 3). Contrary to the usual heterozygote allele-specific dosage patterns found in nearly all triplications (3:1 only or both 3:1 and 2:2 ratios), the CMAs in these cases demonstrate an exclusive 2:2 (AA; BB) allele pattern, indicating that the two extra copies are duplicates from each parental haplotype. Since FISH studies confirmed that the triplication was present in a single homologue with an inverted middle copy, one of the tandem copies had to have originated from the other parental homologue. This genomic rearrangement, has been attributed to mitotic recombination secondary to postzygotic template switching mediated microhomology break induced repair (MMBIR) [20, 32–35]. Our evidence supports the assertion that these rearrangements arise through correction of the relatively common class of meiotic rearrangements with inverted duplications contiguous with terminal deletions [36, 37]. This mechanism is supported by the mosaicism detected in an analysis of a fetal loss, in which an intermediate allele dosage plot suggested a mixture of two cell lines: one with an inverted duplication contiguous with a terminal deletion of 8p and the other, in which a triplication and terminal segUPD replaced the duplication and deletion (Case 46). Since the distal end of the duplication is inverted relative to the normal homologue, it is reasonable that microhomology mediates homologue strand annealing, which has been shown by breakpoint junction sequencing [33, 38] between the normal homologue and the distal end of the inverted duplication (Fig. 16). This would provide a telomere necessary for chromosome stability and provide a more viable deletion correction at the expense of adding an additional copy of the duplicated region.

### Mosaic SegUPD

Mosaic segUPD is indicated by the presence of a subpopulation of cells with homozygosity within a specific terminal region (Fig. 3D) that arises in post-zygotic development. Expansion of a segUPD cell population may be random, secondary to an early developmental origin with no apparent advantage over the original normal cells. Alternatively, the cell population may be expanding through an advantage provided by a “driver” gene rendered homozygous in the segUPD interval, as in BWS.

Five cases in this cohort (cases 17, 21, 22, 32, and 33) have centromeric heterochromatin mitotic recombination initiation sites that have been considered predisposed to breakage, which may account for the frequency seen in this study. A break at these sites may be an early somatic event involving loss of the whole arm. The mosaicism may be associated with correction of an early post-zygotic deletion with rapid loss of the deleted cell line. This would make clinical effects less likely, consistent with the lack of correlation of any of these cases with region-specific deletion symptoms.

Cases 8, 24 and 34 provide evidence for an expanding homozygous population. The > 26 Mb region terminal 7q region detected in case 8 spanned too many genes to attempt to ascertain if there was a gene conversion “driver” mutation that might be linked to the cardiac defect observed. Gene sequencing would be very helpful in this type of study. Case 34, which showed two cell populations with distinct mitotic recombination sites and apparent segUPD on the long arm of chromosome 19, contained the *POLD1* gene in the segUPD region, which is a candidate gene for the progeria-like phenotype [39, 40]. The progressive symptoms appear consistent with a selective somatic drive to an abnormal phenotype. Similar selective mechanisms have been shown in patients with Proteus syndrome and related disorders, in which somatic mutations within the *AKT* gene, as well as other genes within the *PI3-AKT* pathway, confer a growth advantage [41]. There is ample precedent for this in cancer CMAs, which frequently show clonal evolution with homozygous conversions of heterozygous mutations [21]. Multiple subclones that exhibit independent initiation sites in the same chromosomal arm proximal to the mutation are also common. Single gene selection to a normal phenotype may be found in patients exhibiting ichthyosis with mottling due to dominant *KRT1* and *KRT10* mutations, in which numerous clones of normal skin arise because of increased viability of cells that have converted to the homozygous wild type gene [23, 25]. These reports show that each of the normal skin foci demonstrated a different mitotic recombination initiation site proximal to the mutation in the chromosome arm, consistent with a high potential for numerous recombination events and little or no site bias.

**Fig. 19:**
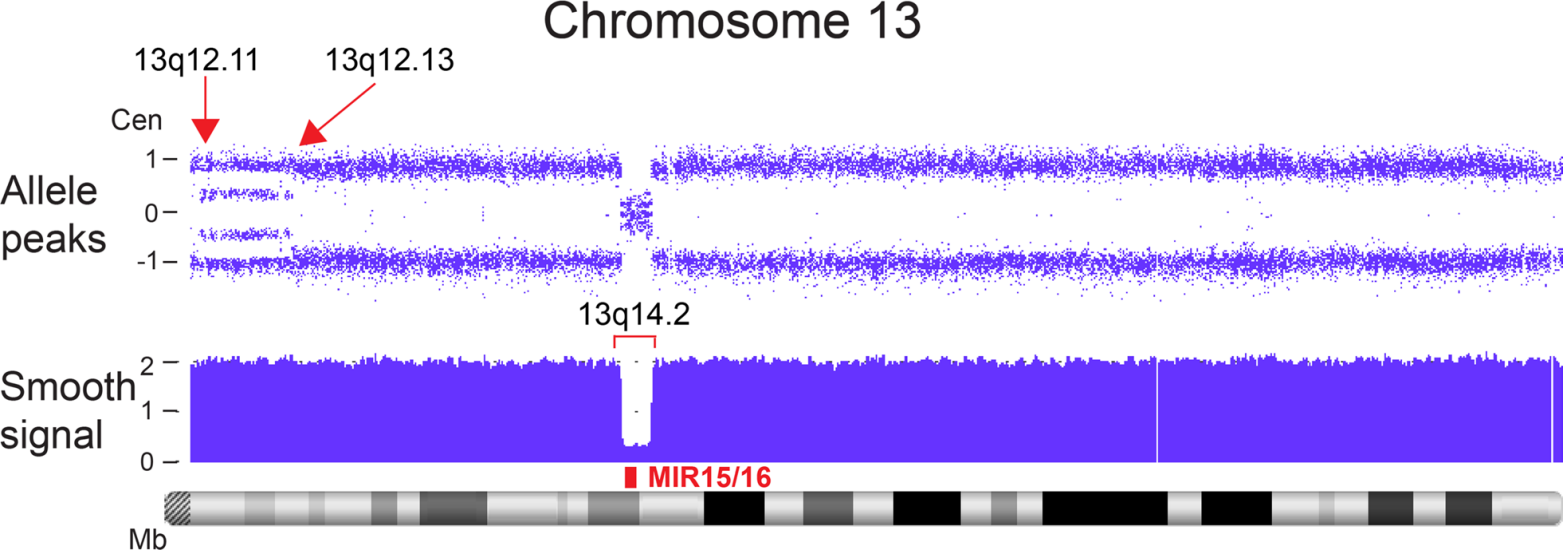
Example of multiple sites of mitotic recombination in clonal based selection in cancer. Archival sample of a leukemia case showing acquired regions of expanding homozygosity referred to as copy neutral loss of heterozygosity (CN-LOH). Two mosaic contiguous regions of CN-LOH (arrows) on chromosome 13, consistent with the evolution of two cells lines with mitotic recombination mediated homozygosity initiated at 13q12.11 and 13q12.13. The selection of recombinant cells is driven by conversion of a heterozygous deletion of the MIR15/16 tumor suppressor genes, red rectangle) at13q14.2 to homozygosity (bracket). A similar pattern is seen in the conversion to normal in case 30 (Fig. 12)

Case 30 showed apparent rescue of a mosaic interstitial deletion of 15q by the concomitant presence of three distinct cell populations with differing levels of homozygosity (Fig. 12). The percentages of residual deletion (~ 25%) and segUPD lines (~ 75%) suggest concurrent selection that resulted in the partial replacement of the original deletion line through ongoing somatic selective pressure for repair. This is analogous to a blood CMA from a patient with chronic lymphocytic leukemia (Fig. 19), in which a deletion of the *MIR15/16* tumor suppressor has evolved to a homozygous loss by positive selection for two independent cell lines with separate mitotic recombination initiation sites resulting in bi-allelic loss. The genes responsible for the selection in case 30 are likely to be within the 15q25.2 microdeletion syndrome critical region at the center of the deletion interval. The proband phenotype is consistent with sustained microdeletion syndrome effects [42].

### Diagnostic and clinical considerations

This study has numerous important implications for clinical diagnoses including: (1) terminal ROH associated with segUPD can arise secondarily to a variety of genomic imbalances, some of which may also have corrected in a carrier parent; (2) corrections are mediated by mitotic recombination that occurs at or proximal to the site of imbalance; (3) clinical effects in corrections of meiotic-derived imbalance are likely to depend on whether the imbalance was sustained in critical tissues during development, making the effects difficult to predict. Additionally, (4) studies of multiple tissues (e.g. buccal cells in addition to blood) may be required to reveal sustained imbalance; (5) mosaic segUPD cases that have progressive symptoms or can be shown to have arisen post-zygotically appear to be more likely mediated by gene-related homozygotic allele advantage; (6) it is likely that at least some cases of reproductive recurrence of the same alteration can be attributed to a parent with abnormal gametes that failed to correct; (7) there appear to be regional “hot spots” for corrections such as 1p and 11q, although evidence of this was not apparent in the UPD database [17]. The lack of examples in the database may be due to the bias for ascertainment of the reported cases for imprinted syndromes and recessive disorders with non-Mendelian inheritance. Our ascertainment is based on the presence of a terminal ROH, which can be associated with a variety of clinical symptoms or an alteration detected in a different tissue or at a previous time in development.

Unfortunately, extended terminal ROH associated with segUPD may be reported as possible identity by descent or not noted at all if below the reporting criteria. A 5 Mb reporting threshold has been recommended [12]. Therefore, terminal ROH should be considered for possible segUPD at sizes smaller than other reporting criteria and with appropriate molecular follow-up testing. Currently, whole exome sequencing methodologies aren’t widely utilized for the detection of segUPD, but extended terminal allele homozygosity should be considered for follow-up trio analysis to effectively rule out segUPD by either whole exome sequencing or CMAs. That testing could be restricted to apparent hot spots like 1p and 11q that are known to have high frequencies of syndrome breakage [43, 44], terminal ROH greater than ~ 5 Mb, and cases without obvious identity by descent. The frequency of segUPD is not known, but mosaic segUPD in younger individuals has been estimated at almost 0.5% [45, 46]. Recent sequencing studies have shown an overall prevalence of whole chromosome UPD as high as 1 in 176 in patients with developmental delay and 1 in 2000 of general population live births [8, 9]. It is likely that the incidence of these cases will increase since NIPT studies are able to detect early evidence of imbalances, which in some cases, may be subsequently corrected leaving the associated terminal ROH. These cases present genetic counseling challenges because it will not be known whether the original imbalance will be associated with clinical effects in the fetus or the correction has occurred early enough to offer a complete clinical rescue.

Clearly, NIPT results that are discordant with subsequent testing provide an excellent opportunity to identify dynamic placental-fetal corrections. We have recently reported 3 additional discordant cases associated with segUPD [47]. Many of these aberrations are restricted to the placenta, but some also show residual fetal mosaicism in follow-up analyses.

These examples of NIPT genomic corrections indicate that changes may need to be made in parental follow-up testing. We propose that following normal parental follow-up karyotyping (consistent with a de novo rearrangement in their child), SNP microarray testing could be performed in the region of imbalance to exclude segmental UPD in the parents and the associated high risk of recurrence for accurate prenatal counseling. If half the oocytes in a female with segUPD associated rescue of a meiotic genomic imbalance are abnormal, the risk of recurrence in future offspring may be as high as 50%. It is possible that paternally derived imbalances may not be subject to germ cell correction exclusion as with oocytes. Further studies are necessary to determine the overall incidence of corrections, to confirm hot spots for corrections such as the 1p and 11q terminal regions, and to ascertain if paternal germ cells have the potential to correct. Selection-based somatic recombination-mediated repair may be a relatively frequent occurrence with patent reproductive risk and may explain the etiology of clinical phenotypes in more patients than currently known.

## Supplementary Information

**Additional file 1.** **Figure S1**. SegUPD parent of origin study of case 1. **Figure S2**. SegUPD parent of origin study of case 6. **Figure S3**. SegUPD parent of origin study of case 9. **Figure S4**. SegUPD parent of origin study of case 12 (A: Microarray indicating large ROH on chromosome 15; B: SNP analysis confirming paternal segUPD of q15.3->qter).

## Data Availability

Tables and supplementary data are included in the manuscript.

## Abbreviations

AF: Amniotic fluid
AMA: Advanced maternal age
arr: Microarray
AV: Atrioventricular
BWS: Beckwith–Wiedemann syndrome
Cen: Centromere
cfDNA: Cell-free deoxyribonucleic acid (DNA)
CMA: Chromosomal microarray analysis
CN-LOH: Copy-neutral loss of heterozygosity
CVS: Chorionic villus sample
der: Derivative
DNA: Deoxyribonucleic acid
EEG: Electroencephalogram
FISH: Fluorescence in situ hybridization
JS: Jacobsen syndrome
Mat: Maternal
Mb: Megabases
MMBIR: Microhomology break induced repair
MSS: Maternal serum screening
NA: Not available
NB: Newborn
NIPT: Non-invasive prenatal testing
NT: Nuchal translucency
Pat: Paternal
POC: Products of conception (tissue)
pter: p arm terminus
qter: q arm terminus
ROH: Run(s) of homozygosity
segUPD: Segmental uniparental disomy
SNP: Single nucleotide polymorphism
t: Translocation
Tel: Telomere
UPD: Uniparental disomy
VSD: Ventricular septal defect

## References

[1] Engel E (1980). A new genetic concept: uniparental disomy and its potential effect, isodisomy. Am J Med Genet.

[2] Papenhausen P, Schwartz S, Risheg H, Keitges E, Gadi I, Burnside RD, Jaswaney V, Pappas J, Pasion R, Friedman K, Tepperberg J (2011). UPD detection using homozygosity profiling with a SNP genotyping microarray. Am J Med Genet A.

[3] Kearney HM, Kearney JB, Conlin LK (2011). Diagnostic implications of excessive homozygosity detected by SNP-based microarrays: consanguinity, uniparental disomy, and recessive single-gene mutations. Clin Lab Med.

[4] Lapunzina P, Monk D (2011). The consequences of uniparental disomy and copy number neutral loss-of-heterozygosity during human development and cancer. Biol Cell.

[5] Schollen E, Grünewald S, Keldermans L, Albrecht B, Körner C, Matthijs G (2005). CDG-Id caused by homozygosity for an ALG3 mutation due to segmental maternal isodisomy UPD3 (q21. 3-qter). Eur J Med Genet.

[6] Robinson WP (2000). Mechanisms leading to uniparental disomy and their clinical consequences. BioEssays.

[7] Scuffins J, Keller-Ramey J, Dyer L, Douglas G, Torene R, Gainullin V, Juusola J, Meck J, Retterer K (2021). Uniparental disomy in a population of 32,067 clinical exome trios. Genet Med.

[8] Nakka P, Smith SP, O’Donnell-Luria AH, McManus KF, Agee M, Auton A, Bell RK, Bryc K, Elson SL, Fontanillas P, Furlotte NA (2019). Characterization of prevalence and health consequences of uniparental disomy in four million individuals from the general population. Am J Hum Genet.

[9] Yauy K, de Leeuw N, Yntema HG, Pfundt R, Gilissen C (2020). Accurate detection of clinically relevant uniparental disomy from exome sequencing data. Genet Med.

[10] Cooper WN, Curley R, Macdonald F, Maher ER (2007). Mitotic recombination and uniparental disomy in Beckwith–Wiedemann syndrome. Genomics.

[11] Liehr T (2010). Cytogenetic contribution to uniparental disomy (UPD). Mol Cytogenet.

[12] Hoppman N, Rumilla K, Lauer E, Kearney H, Thorland E (2018). Patterns of homozygosity in patients with uniparental disomy: detection rate and suggested reporting thresholds for SNP microarrays. Genet Med.

[13] Del Gaudio D, Shinawi M, Astbury C, Tayeh MK, Deak KL, Raca G (2020). Diagnostic testing for uniparental disomy: a points to consider statement from the American College of Medical Genetics and Genomics (ACMG). Genet Med.

[14] Moynahan ME, Jasin M (2010). Mitotic homologous recombination maintains genomic stability and suppresses tumorigenesis. Nat Rev Mol Cell Biol.

[15] Eggermann T, Mergenthaler S, Eggermann K, Albers A, Linnemann K, Fusch C, Ranke MB, Wollmann HA (2001). Identification of interstitial maternal uniparental disomy (UPD) (14) and complete maternal UPD (20) in a cohort of growth retarded patients. J Med Genet.

[16] Martin RA, Sabol DW, Rogan PK (1999). Maternal uniparental disomy of chromosome 14 confined to an interstitial segment (14q23–14q24. 2). J Med Genet.

[17] Liehr T. Cases with uniparental disomy. http://cs-tl.de/DB/CA/UPD/0-Start.html. Accessed 04 May 2021.

[18] Jensen TJ, Zwiefelhofer T, Tim RC, Džakula Ž, Kim SK, Mazloom AR, Zhu Z, Tynan J, Lu T, McLennan G, Palomaki GE (2013). High-throughput massively parallel sequencing for fetal aneuploidy detection from maternal plasma. PLoS ONE.

[19] Lefkowitz RB, Tynan JA, Liu T, Wu Y, Mazloom AR, Almasri E, Hogg G, Angkachatchai V, Zhao C, Grosu DS, McLennan G (2016). Clinical validation of a noninvasive prenatal test for genomewide detection of fetal copy number variants. Am J Obstet Gynecol.

[20] Papenhausen PR, Kelly CA, Zvereff V, Schwartz S (2014). Four-copy number intervals in SNP microarray analysis: unique patterns and positions. Cytogenet Genome Res.

[21] O'Keefe C, McDevitt MA, Maciejewski JP (2010). Copy neutral loss of heterozygosity: a novel chromosomal lesion in myeloid malignancies. Blood.

[22] Cajaiba MM, Witchel S, Madan-Khetarpal S, Hoover J, Hoffner L, Macpherson T, Surti U (2011). Prenatal diagnosis of trisomy 6 rescue resulting in paternal UPD6 with novel placental findings. Am J Med Genet A.

[23] Choate KA, Lu Y, Zhou J, Choi M, Elias PM, Farhi A, Nelson-Williams C, Crumrine D, Williams ML, Nopper AJ, Bree A (2010). Mitotic recombination in patients with ichthyosis causes reversion of dominant mutations in KRT10. Science.

[24] Kiraly O, Gong G, Olipitz W, Muthupalani S, Engelward BP (2015). Inflammation-induced cell proliferation potentiates DNA damage-induced mutations in vivo. PLoS Genet.

[25] Choate KA, Lu Y, Zhou J, Elias PM, Zaidi S, Paller AS, Farhi A, Nelson-Williams C, Crumrine D, Milstone LM, Lifton RP (2015). Frequent somatic reversion of KRT1 mutations in ichthyosis with confetti. J Clin Investig.

[26] dos Santos A, Campagnari F, Krepischi ACV, Ribeiro Câmara ML, de Arruda Brasil RDCE, Vieira L, Vianna-Morgante AM, Otto PA, Pearson PL, Rosenberg C (2018). Insight into the mechanisms and consequences of recurrent telomere capture associated with a sub-telomeric deletion. Chromosom Res.

[27] Cohen AS, Wilson SL, Trinh J, Ye XC (2015). Detecting somatic mosaicism: considerations and clinical implications. Clin Genet.

[28] Kucińska-Chahwan A, Bijok J, Dąbkowska S, Jóźwiak A, Ilnicka A, Nowakowska B, Jakiel G, Roszkowski T (2017). Targeted prenatal diagnosis of Pallister–Killian syndrome. Prenat Diagn.

[29] Johnson JP, Haag M, Beischel L, McCann C, Phillips S, Tunby M, Hansen J, Schwanke C, Reynolds JF (2014). ‘Deletion rescue’ by mitotic 11q uniparental disomy in a family with recurrence of 11q deletion Jacobsen syndrome. Clin Genet.

[30] Kawai M, Tsutsumi M, Suzuki F, Sameshima K, Dowa Y, Kyoya T, Inagaki H, Kurahashi H (2019). Two siblings with 11qter deletion syndrome that had been rescued in their mother by uniparental disomy. Eur J Med Genet.

[31] Afifi HH, Zaki MS, El-Gerzawy AM, Kayed HF (2008). Distal 11q monosomy syndrome: a report of two Egyptian sibs with normal parental karyotypes confirmed by molecular cytogenetics. Genet Couns.

[32] Bonaglia MC, Giorda R, Beri S, Bigoni S, Sensi A, Baroncini A, Capucci A, De Agostini C, Gwilliam R, Deloukas P, Dunham I (2009). Mosaic 22q13 deletions: evidence for concurrent mosaic segmental isodisomy and gene conversion. Eur J Hum Genet.

[33] Carvalho CM, Pfundt R, King DA, Lindsay SJ, Zuccherato LW, Macville MV, Liu P, Johnson D, Stankiewicz P, Brown CW, Study DD (2015). Absence of heterozygosity due to template switching during replicative rearrangements. Am J Hum Genet.

[34] Sahoo T, Wang JC, Elnaggar MM, Sanchez-Lara P, Ross LP, Mahon LW, Hafezi K, Deming A, Hinman L, Bruno Y, Bartley JA (2015). Concurrent triplication and uniparental isodisomy: evidence for microhomology-mediated break-induced replication model for genomic rearrangements. Eur J Hum Genet.

[35] Kohmoto T, Okamoto N, Naruto T, Murata C, Ouchi Y, Fujita N, Inagaki H, Satomura S, Okamoto N, Saito M, Masuda K (2017). A case with concurrent duplication, triplication, and uniparental isodisomy at 1q42. 12-qter supporting microhomology-mediated break-induced replication model for replicative rearrangements. Mol Cytogenet.

[36] Hermetz KE, Newman S, Conneely KN, Martin CL, Ballif BC, Shaffer LG, Cody JD, Rudd MK (2014). Large inverted duplications in the human genome form via a fold-back mechanism. PLoS Genet.

[37] Zuffardi O, Bonaglia M, Ciccone R, Giorda R (2009). Inverted duplications deletions: underdiagnosed rearrangements?. Clin Genet.

[38] Kato T, Inagaki H, Miyai S, Suzuki F, Naru Y, Shinkai Y, Kato A, Kanyama K, Mizuno S, Muramatsu Y, Yamamoto T (2020). The involvement of U-type dicentric chromosomes in the formation of terminal deletions with or without adjacent inverted duplications. Hum Genet.

[39] Shastry S, Simha V, Godbole K, Sbraccia P, Melancon S, Yajnik CS, Novelli G, Kroiss M, Garg A (2010). A novel syndrome of mandibular hypoplasia, deafness, and progeroid features associated with lipodystrophy, undescended testes, and male hypogonadism. J Clin Endocrinol Metab.

[40] Weedon MN, Ellard S, Prindle MJ, Caswell R, Allen HL, Oram R, Godbole K, Yajnik CS, Sbraccia P, Novelli G, Turnpenny P (2013). An in-frame deletion at the polymerase active site of POLD1 causes a multisystem disorder with lipodystrophy. Nat Genet.

[41] Keppler-Noreuil KM, Parker VE, Darling TN, Martinez-Agosto JA (2016). Somatic overgrowth disorders of the PI3K/AKT/mTOR pathway and therapeutic strategies. Am J Med Genet Part C Semin Med Genet.

[42] Burgess T, Brown NJ, Stark Z, Bruno DL, Oertel R, Chong B, Calabro V, Kornberg A, Sanderson C, Kelly J, Howell KB (2014). Characterization of core clinical phenotypes associated with recurrent proximal 15q25. 2 microdeletions. Am J Med Genet Part A.

[43] Shaffer LG, Lupski JR (2000). Molecular mechanisms for constitutional chromosomal rearrangements in humans. Annu Rev Genet.

[44] Ballif BC, Yu W, Shaw CA, Kashork CD, Shaffer LG (2003). Monosomy 1p36 breakpoint junctions suggest pre-meiotic breakage–fusion–bridge cycles are involved in generating terminal deletions. Hum Mol Genet.

[45] Sasaki K, Mishima H, Miura K, Yoshiura KI (2013). Uniparental disomy analysis in trios using genome-wide SNP array and whole-genome sequencing data imply segmental uniparental isodisomy in general populations. Gene.

[46] Laurie CC, Laurie CA, Rice K, Doheny KF, Zelnick LR, McHugh CP, Ling H, Hetrick KN, Pugh EW, Amos C, Wei Q (2012). Detectable clonal mosaicism from birth to old age and its relationship to cancer. Nat Genet.

[47] Caldwell S, Sagaser K, Nelson Z, Frey J, Wardrop J, Boomer T, McCullough R, Schwartz S (2020). Deletion rescue resulting in segmental homozygosity: a mechanism underlying discordant NIPT results. Am J Med Genet A.

