# Supplementary material for: Clinical significance and mechanisms associated with segmental UPD
Source: Mol Cytogenet. 2021 Jul 20;14:38. doi: 10.1186/s13039-021-00555-0 (PMC8290618; doi:10.1186/s13039-021-00555-0)
Supplement: Supplementary file 1 — Additional file 1. Figure S1. SegUPD parent of origin study of case 1. Figure S2. SegUPD parent of origin study of case 6. Figure S3. SegUPD parent of origin study of case 9. Figure S4. SegUPD parent of origin study of case 12 (A: Microarray indicating large ROH on chromosome 15; B: SNP analysis confirming paternal segUPD of q15.3->qter). [file 13039_2021_555_MOESM1_ESM.docx]

Clinical Significance and Mechanisms Associated with Segmental UPD

Peter R. Papenhausen, ^[[1]](#footnote-1)^ Carla A. Kelly,^1^ Samuel Harris,^[[2]](#footnote-2)^ Samantha Caldwell,^1^ Stuart Schwartz,^1^ Andrea Penton,^1^*

Supplemental data


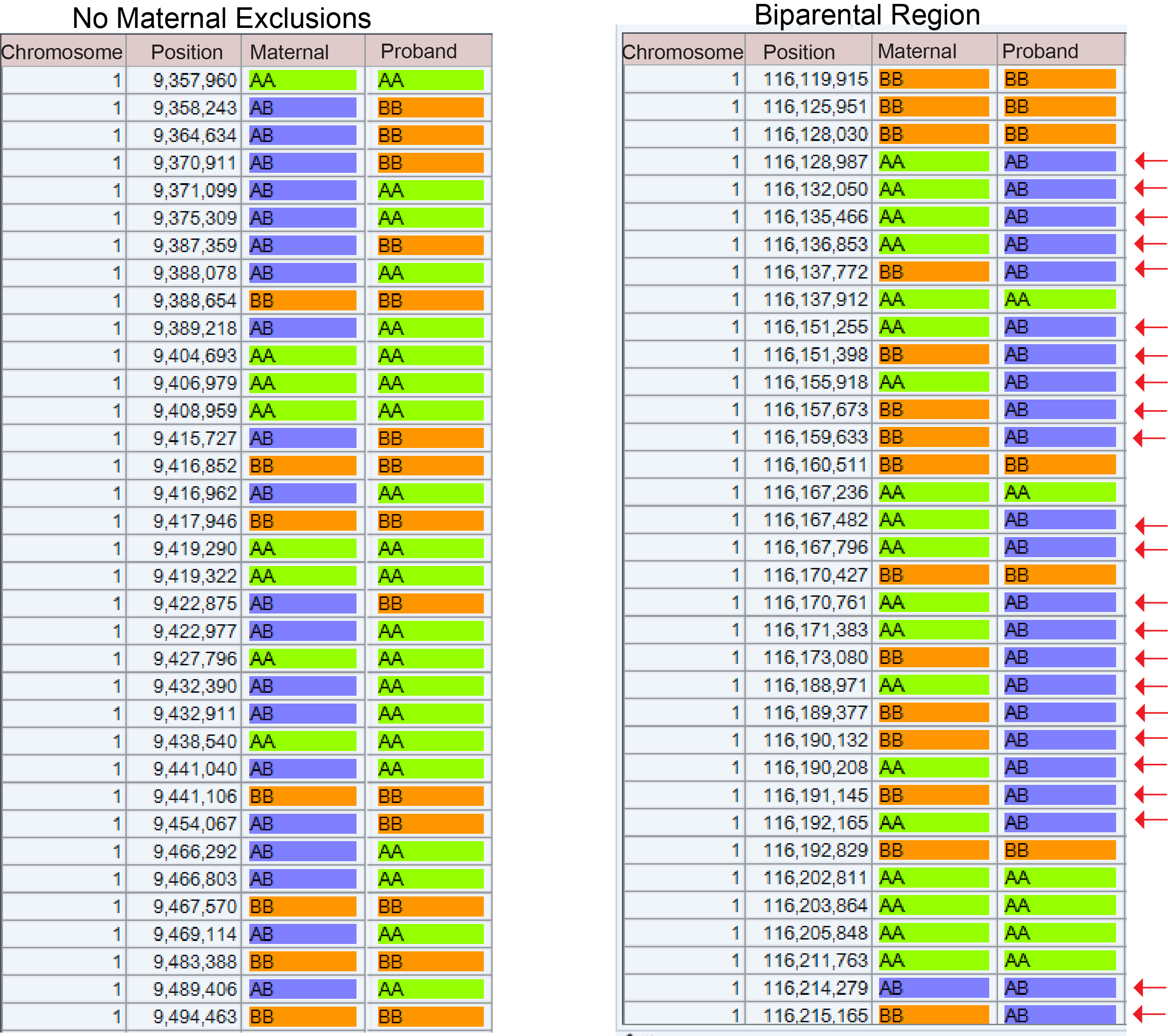


Figure S1**. SegUPD parent of origin study of case 1.** Presumptive maternal segUPD of case 1. No maternal exclusions are detected in hundreds of SNPs in the chromosome 1 terminal ROH (subset shown in figure, left panel) while biparental inheritance is easily confirmed proximal to terminal ROH (biparental SNPs indicated by arrows, right panel).


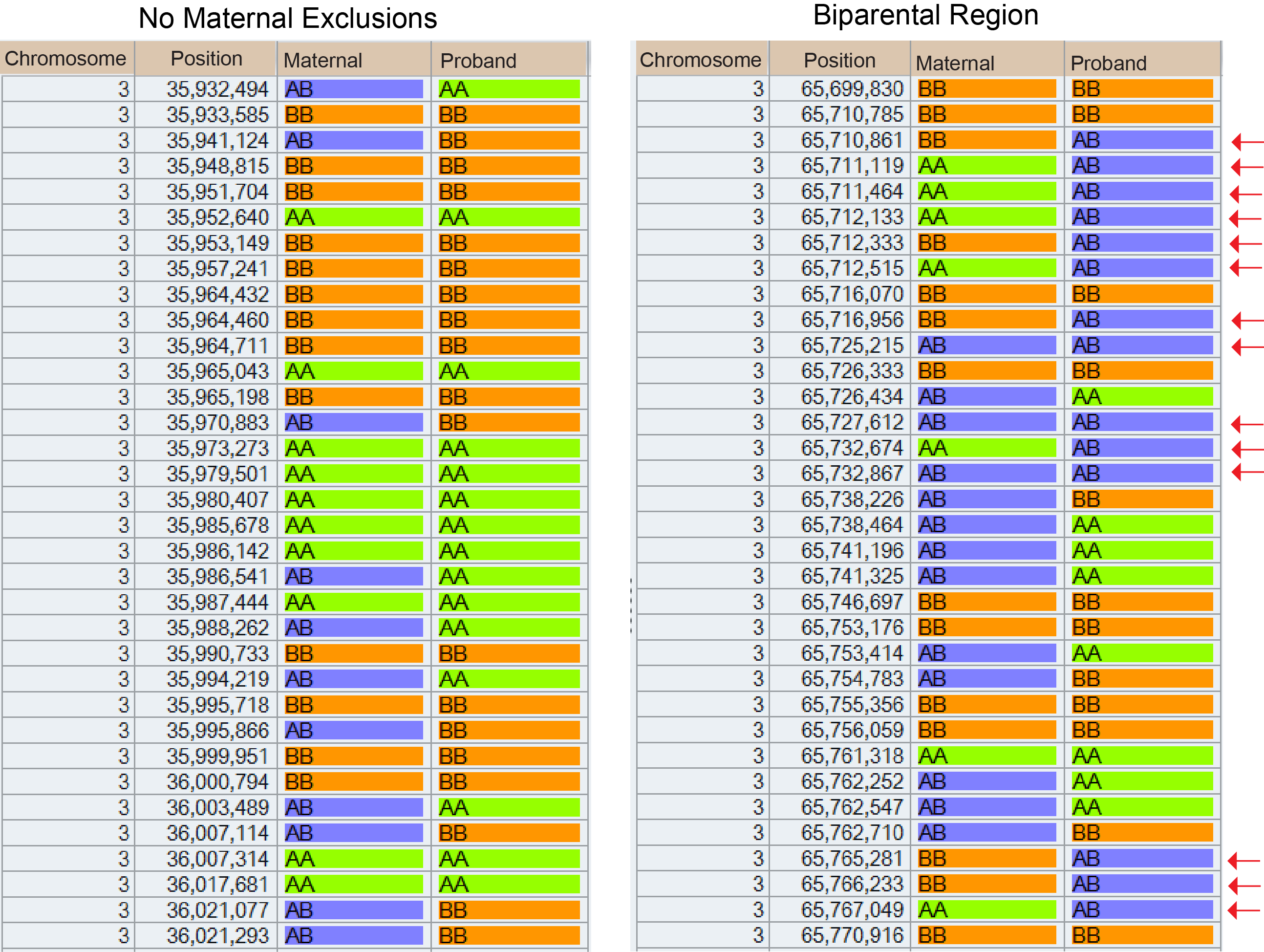


Figure S2. **SegUPD parent of origin study of case 6.** Maternal-proband CMA allele comparison with no maternal exclusions in hundreds of SNPs (left panel) and normal maternal chromosome study confirmed the maternal segUPD and a de novo origin of the derivative 4 in case 6. Biparental inheritance in region proximal to ROH (right panel, biparental SNPs indicated by arrows).


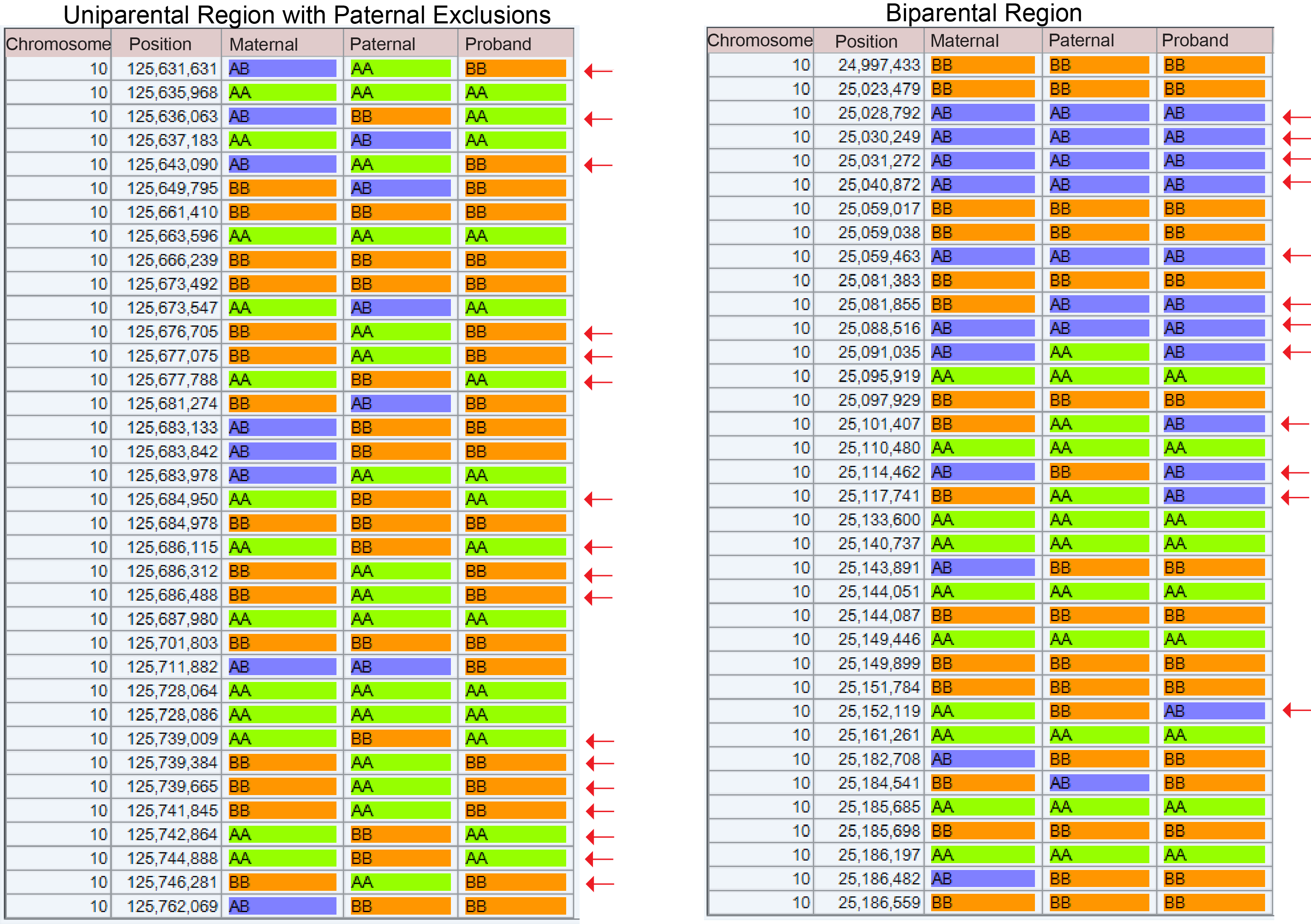


Figure S3. **SegUPD parent of origin study of case 9.** Numerous paternal SNP exclusions (arrows, left panel) restricted to bands q26.13q26.3 within the chromosome 10 ROH confirm maternal segUPD and paternal origin of the deletion of case 9. Biparental inheritance is detected proximally to the terminal ROH (biparental SNPs indicated by arrows, right panel).


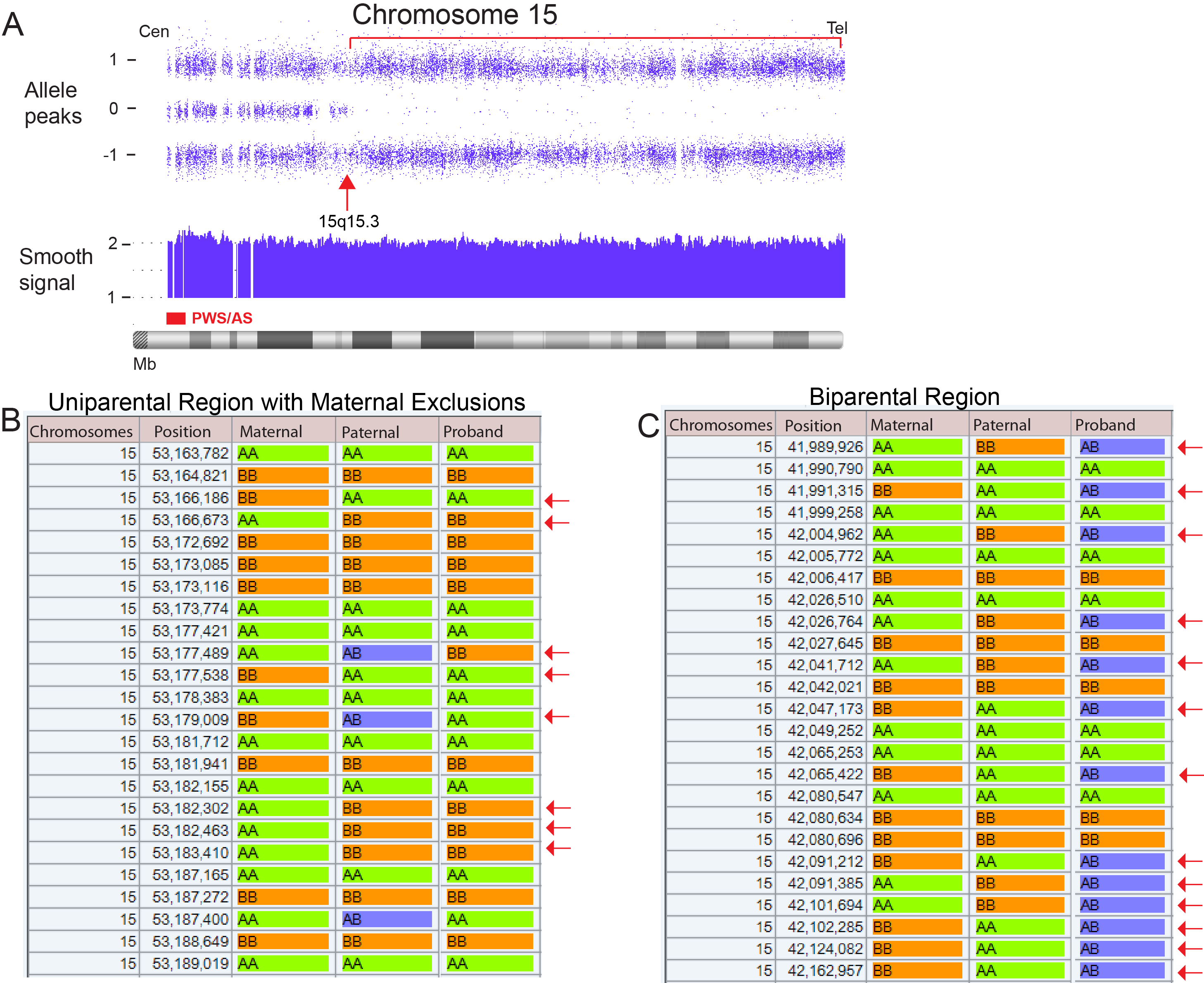


Figure S4. **SegUPD parent of origin study of case 12.** A) Large terminal 58.54 Mb ROH located at bands q15.3→qter of chromosome 15 in case 12. B, C) Numerous maternal SNP exclusions (arrows) confirm paternal segUPD with biparental inheritance detected proximally (biparental SNPs indicated by arrows).

1. Cytogenetics Department, Laboratory Corporation of America, Research Triangle Park, NC 27709, USA [↑](#footnote-ref-1)
2. Morsani College of Medicine, University of South Florida, Tampa, FL 33612, USA

   *Correspondence: pentona@labcorp.com [↑](#footnote-ref-2)
